# Infiltrating peripheral monocyte TREM-1 mediates dopaminergic neuron injury in substantia nigra of Parkinson’s disease model mice

**DOI:** 10.1038/s41419-025-07333-5

**Published:** 2025-01-14

**Authors:** Wei Song, Zi-ming Zhou, Le-le Zhang, Hai-feng Shu, Jin-ru Xia, Xia Qin, Rong Hua, Yong-mei Zhang

**Affiliations:** 1 NMPA Key Laboratory for Research and Evaluation of Narcotic and Psychotropic Drugs, Xuzhou, China; 2 Jiangsu Province Key Laboratory of Anesthesiology, Xuzhou Medical University, Xuzhou, China; 3 Jiangsu Province Key Laboratory of Anesthesia and Analgesia Application Technology, Xuzhou Medical University, Xuzhou, China; 4 Yancheng Stomatological Hospital, Yancheng, China; 5 Department of Emergency, The Affiliated Hospital of Xuzhou Medical University, Xuzhou, China

**Keywords:** Monocytes and macrophages, Neuroimmunology

## Abstract

Neuroinflammation is a key factor in the pathogenesis of Parkinson’s disease (PD). Activated microglia in the central nervous system (CNS) and infiltration of peripheral immune cells contribute to dopaminergic neuron loss. However, the role of peripheral immune responses, particularly triggering receptor expressed on myeloid cells-1 (TREM-1), in PD remains unclear. Using a 1-methyl-4-phenyl-1,2,3,6-tetrahydropyridine hydrochloride (MPTP)-induced PD mouse model, we examined TREM-1 expression and monocyte infiltration in the substantia nigra pars compacta (SNpc). We found that MPTP increased peripheral monocytes, and deletion of peripheral monocytes protected against MPTP neurotoxicity in the SNpc. TREM-1 inhibition, both genetically and pharmacologically, reduced monocyte infiltration, alleviated neuroinflammation, and preserved dopaminergic neurons, resulting in improved motor function. Furthermore, adoptive transfer of TREM-1-expressing monocytes from PD model mice to naive mice induced neuronal damage and motor deficits. These results underscore the critical role of peripheral monocytes and TREM-1 in PD progression, suggesting that targeting TREM-1 could be a promising therapeutic approach to prevent dopaminergic neurodegeneration and motor dysfunction in PD.

**Figure:**
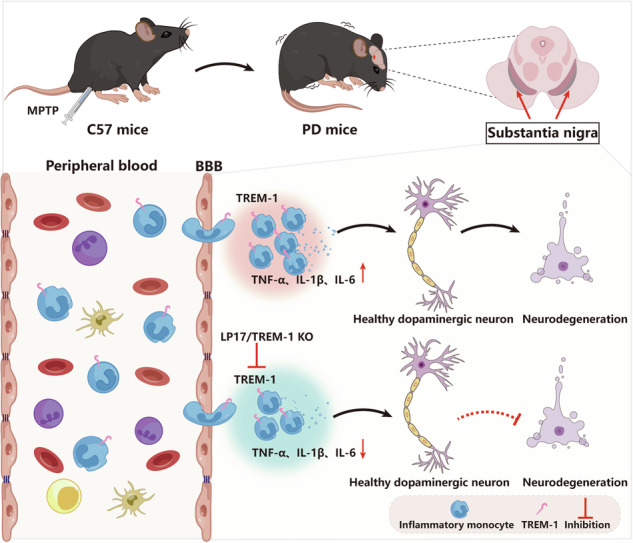
Schematic diagram of monocyte TREM-1-mediated dopaminergic neuron damage. The figure illustrates that in experimental MPTP-induced PD model mice, the number of inflammatory monocytes in the peripheral blood increases, after which the monocytes infiltrate the CNS through the Blood-Brain Barrier(BBB). These infiltrating monocytes increase the release of inflammatory cytokines and eventually cause neuronal injury. TREM-1 gene deletion and pharmacological blockade limit inflammatory monocyte recruitment into the SNpc and ameliorate neuroinflammatory events and the loss of dopaminergic neurons.

Schematic diagram of monocyte TREM-1-mediated dopaminergic neuron damage. The figure illustrates that in experimental MPTP-induced PD model mice, the number of inflammatory monocytes in the peripheral blood increases, after which the monocytes infiltrate the CNS through the Blood-Brain Barrier(BBB). These infiltrating monocytes increase the release of inflammatory cytokines and eventually cause neuronal injury. TREM-1 gene deletion and pharmacological blockade limit inflammatory monocyte recruitment into the SNpc and ameliorate neuroinflammatory events and the loss of dopaminergic neurons.

## Introduction

Parkinson’s disease (PD) is the second most common neurodegenerative disorder. The pathological hallmark of PD is the progressive loss of dopaminergic neurons in the substantia nigra pars compacta (SNpc) [1], which results in extrapyramidal system dyskinesia accompanied by manifestations of resting tremor, rigidity, and postural bradykinesia. Although the pathogenesis of PD is poorly understood, mounting evidence indicates that inflammation plays a crucial role in the development of PD [2, 3]. The central nervous system (CNS) was previously considered an immune-privileged system, but in recent years, it has become increasingly evident that the CNS communicates extensively with the peripheral immune system [4]. Numerous studies support a deleterious role of peripheral inflammation in PD, such as elevated levels of inflammatory cytokines in body fluids [5–7] and aberrant functions and proportions of lymphocytes [8, 9] and monocytes [10–12]. Myeloid cells, including monocytes, are the pivotal regulatory cells of the immune system [13]. Considering the various phenotypes of monocytes at different states, the exact role of monocytes during PD may differ. Under physiologically normal conditions, the CNS is isolated from the periphery by the blood-brain barrier (BBB), and monocytes may also gain access to the brain parenchyma under certain disease conditions [14]. Immediately after the BBB is compromised, monocytes migrate into the brain parenchyma and differentiate into macrophages to mediate pro and anti-inflammatory responses [15–17].

Triggering receptor expressed on myeloid cells-1 (TREM-1), which is prominently expressed on the surface of neutrophils, subsets of monocytes, and macrophages, functions as an inflammation amplifier and plays a vital role in innate and adaptive immunity [18]. Under diverse inflammatory conditions, the expression of TREM-1 is upregulated [19–21]. This TREM-1-mediated enhancement of the proinflammatory immune response has been demonstrated in several models of infection, inflammatory bowel disease [20, 22, 23], septic shock [24], and sepsis syndrome [25], it is also critical in some sterile inflammatory conditions, including atherosclerosis [26], postischemic myocardial remodeling [27], abdominal aortic aneurysm [28], and rheumatoid arthritis [29]. Previous studies have shown that therapeutic inhibition of TREM-1 can blunt excessive inflammatory cell infiltration, resulting in a decreased severity of liver injury [30] and abdominal aortic aneurysm [28].

In the present study, we explored the new role of TREM-1 in a mouse model of PD. We presented novel findings regarding the impact of TREM-1 gene deletion and pharmacological blockade on the recruitment of Ly6C^hi^ classical inflammatory monocytes into the SNpc and the subsequent attenuation of dopaminergic neuron loss. Thus, our study underscores the contribution of peripheral inflammation to the loss of dopaminergic neurons in PD model mice and identifies monocyte TREM-1 as a new factor in the pathophysiology of PD, which may constitute a novel systemic therapy for PD patients.

## Materials and methods

### Animals

Male TREM-1 knockout (B6/JGpt-Trem1^em1Cd6026^6026/Gpt) mice (weighing 25–30 g, 8 weeks old) on a C57BL/6 J genetic background were generated by Gempharmatech Corporation (Nanjing, China). Male wild-type (WT) C57BL/6 J mice (weighing 25–30 g, 8 weeks old) were purchased from Changzhou Cavens Laboratory Animal Corporation (Changzhou, China). All mice were housed in a temperature-controlled room (at 22 ± 1 °C and 40–60% relative humidity), with a 12/12-h light/dark cycle, and permitted free access to food and water. In mice experiment, no statistical methods were used to predetermine sample size. For morphological and immunofluorescence analyses, a sample size of 3–6 mice was used. For behavioral experiments, the sample size ranged from 6 to 16 mice, depending on the specific test. We assigned mice to experimental groups arbitrarily, without randomization or blinding.

### Ethics approval and consent to participate

All animal experiments were performed in accordance with the National Institutes of Health Guide for the Care and Use of Laboratory Animals. The protocol was preapproved by the Institutional Animal Care and Use Committee of Xuzhou Medical University (approval No. 202010A017).

### Drugs and treatments

For MPTP intoxication, C57BL/6 J mice received intraperitoneal injections of MPTP (30 mg/kg) (MedChemExpress, Shanghai, China) dissolved in 0.9% saline on 5 consecutive days. The control mice were injected with an equivalent volume of saline only; For pharmacological blockade of TREM-1, as previously described [31], the TREM-1 blocking peptide LQVTDSGLYRCVIYHPP (LP17) was chemically synthesized by GenScript (Nanjing, China). Starting on Day 0 (at the beginning of MPTP injection), the mice were treated with either an LP17 peptide or a sequence-scrambled control peptide TDSRCVIGLYHPPLQVY. Given the short half-life of peptides in vivo, mice were treated once daily with 1 mg/kg peptide and administered intranasally in 200 μl of saline [32]. For the monocyte depletion experiments, the mice were treated as follows. Monocytes were depleted by tail vein injection of 200 μl of clodronate liposome (CLP) or PBS liposomes into each mouse every 2 days. These mice were sacrificed at 7 days after the first MPTP injection.

### Antibodies and chemicals

All primary and secondary antibodies used in the present study are listed in Tables 1 and 2. BCA protein assay kits were from Beyotime (P0012, China). 1-methyl-4-phenylpyridinium (MPP) ion MPP^+^ (36913-39-0) and dopamine hydrochloride (62-31-7) standards (with purities higher than 95% according to HPLC) were purchased from Weikeqi Biotech (China). LP17 (887255-16-5) standards (with purities higher than 95% according to HPLC) were purchased from Macklin Biochemical (China).

**Table 1:** Primary antibodies.

Name of antibody	Host	Fluorochrome	Manufacturer, catalog number	Application	Working dilutions or concentrations
CD45	Mouse	APC	Biolegend, 103112	Flow Cytometry	1:200
CD11b	Mouse	FITC	Biolegend, 101206	Flow Cytometry	1:500
Ly6G	Mouse	PE	Biolegend, 127608	Flow Cytometry	1:200
Ly6G	Mouse	Percp-cy5.5	Biolegend, 127616	Flow Cytometry	1:200
Ly6C	Mouse	Percp-cy5.5	Biolegend, 128012	Flow Cytometry	1:200
Ly6C	Mouse	BV605	Biolegend, 128035	Flow Cytometry	1:200
CX3CR1	Mouse	PE-cy7	Biolegend, 149016	Flow Cytometry	1:200
TREM-1	Mouse	PE	Invitrogen, MA5-28221	Flow Cytometry	1:400
Iba1	Mouse	-	Wako, 019-19741	IF	1:500
Hexb	Rabbit	-	Proteintech, 16229-1-AP	IF	1:200
TH	Mouse	-	Abcam, ab217161	IF	1:400
				WB	1:2000
TNF-α	Rabbit	-	Proteintech, 1590-1-AP	WB	1:1000
TREM-1	Mouse	-	Novusbio	WB	1:500
IL-1β	Rabbit	-	Proteintech, 26048-1-AP	WB	1:1000
IL-6	Rabbit	-	Proteintech, 29444-1-AP	WB	1:1000
β-actin	Rabbit	-	Proteintech, 66009-1-Ig	WB	1:2000
Tubulin	Rabbit	-	Affinity, DF7967	WB	1:3000

**Table 2:** Secondary antibodies.

Antibody	Host	Manufacturer	Catalog number	Application	Working dilutions or concentrations
Anti-rabbit IgG, HRP	Goat	Proteintech	SA00001-2	WB	1:2000
Anti-mouse IgG, HRP	Goat	Proteintech	SA00001-1	WB	1:2000
Anti-rabbit IgG, Alexa 594	Donkey	Invitrogen	A21207	IF	1:600
Anti-mouse IgG, Alexa 488	Donkey	Invitrogen	A21202	IF	1:400

### Behavior tests

#### Open field test (OFT)

Motor behavior was analyzed in an open field test 5 days after intraperitoneal injection of MPTP. The open field device consisted of a square area with a surrounding wall. Approximately 1 h before the experiment, the mice were transferred to the laboratory for adaptation. Mice (n = 8–16 per group) were then placed into the center of an open field device with evenly distributed light for 5 minutes. During the test, the total distance traveled was automatically recorded by ANY-Maze software over a 5-min period.

#### Rotarod test

Mouse motor coordination was assessed by the rotarod test as previously described [33]. The training was carried out for 3 consecutive days before the administration of MPTP until no fall was detected within 300 s. Mice (n = 6–8 per group) were gently returned to the rod during training if they slipped off. During the experiment, all the mice walked on the rod steadily from 5 rpm to 40 rpm in 300 s. The latency to fall off the rod was recorded up to a maximum of 300 s.

#### Pole test

To evaluate the severity of bradykinesia, the pole test was performed according to previous methods [34]. The mice were (n = 6–10 per group) trained three times to correctly descend from the top to the bottom of the pole (75 cm in length and 1 cm in diameter) before the establishment of the model. The time needed to reach the bottom of the pole was recorded during the test. The mice were subjected to three trials at 30-minute intervals. The results from three trials were averaged.

#### Immunofluorescence (IF)

After the behavioral tests, whole-brain tissues were collected, perfused with 4% paraformaldehyde for 24 h, and subsequently dehydrated with 30% sucrose solution for 48 h. Thirty-micron-thick coronal sections containing the SNpc were collected with a freezing microtome (CM1800, Leica, Germany) for immunofluorescence staining. The cryosections were washed with 0.1% Triton X-100 in PBS for 10 min and blocked with 10% goat serum for 1 h at room temperature (RT). The sections were then incubated with primary antibodies against tyrosine hydroxylase (TH), Iba-1, and hexosaminidase subunit beta (Hexb) overnight at 4 °C. The sections were then incubated with the appropriate secondary antibodies. DAPI (Beyotime, China) was used to stain the cell nuclei. Images were taken with a fluorescence microscope (Olympus, Tokyo, Japan). Immunofluorescence revealed that dopaminergic neurons in the SNpc were visible in different groups. Density analysis was performed using previously reported methods [35]. Briefly, we counted the mean number of TH-positive neurons in three consecutive SNpc sections per mouse (n = 9–12 sections/3–4 mice per group) via light microscopy. Using ImageJ software, the total area of the SNpc was obtained, and the density of dopaminergic neurons in the SNpc was subsequently calculated as the number of TH-positive neurons per area (mm^2^), quantified using ImageJ for each field and each section. Two blinded observers assessed each section manually and then the results were used for statistical analyses.

#### Western blot (WB)

Whole SNpc tissues extracted from brains were homogenized in RIPA lysis buffer supplemented with protease inhibitors. After 15 minutes of centrifugation at 12,000 rpm at 4 °C, the supernatant (namely, the total protein) was collected. A BCA protein assay kit (Beyotime, China) was used to determine the protein concentrations. Equal amounts of precipitated protein samples (n = 3–6 per group) were loaded, separated by SDS–PAGE, transferred onto the same PVDF membrane, and blocked for 1 h at RT with 5% milk in Tris-buffered saline with 0.1% Tween-20 (TBST). Then, the membranes were incubated with different primary antibodies against TH, TREM-1, interleukin-6 (IL-6), interleukin-1β (IL-1β), and tumor necrosis factor-α (TNF-α) overnight at 4 °C. After washing, the membranes were incubated with HRP-conjugated secondary antibodies at RT for 1 h. The target protein signal was detected and digitized using an enhanced chemiluminescence system (Bio-Rad, USA). Densitometric quantification of the bands was performed with ImageJ software (NIH, USA).

#### Quantitative reverse transcription polymerase chain reaction (qRT-PCR)

Fresh mouse SNpc was extracted and placed in a nuclease-free Eppendorf (EP) tube containing an appropriate amount of lysate. The homogenate was thoroughly sonicated on ice, and total RNA was extracted from the nigra according to the instructions of the RNA purification kit (Sangon Biotech, China). The nucleic acid concentration of the RNA was measured using the HiScript Q RT SuperMix for qPCR (+gDNA wiper) kit (Vazyme, China) to prepare a reverse transcription reaction solution and using a reverse transcription instrument to reverse record the RNA preparation into cDNA. Using cDNA products as templates, real-time PCR amplification of cDNA was performed using specific primers and ChamQ Universal SYBR qPCR Master Mix (Vazyme, China) reagent on a Thermo Fly QuantStudio 7 Flex (n = 4–6 per group).

The reaction conditions were as follows: 95 °C for 30 seconds, 60 °C for 30 seconds, 72 °C for 60 seconds, and 60 °C for 60 seconds for 40 cycles. Melting curve analysis was performed to determine the specificity of the amplified products. All the reactions contained the same amount of cDNA. The CT method (2^−^^△△Ct^) was used to measure the relative expression of IL-6, IL-1β, and TNF-α, which was normalized to the expression of the β-actin and Gapdh genes.

TREM-1; Forward primer (5’–3’): CCCTGGTGGTCACACAGAG, Reverse prime (5’–3’): GCCTCACTAGGGTCATGTTTC

IL-6; Forward primer (5’–3’): ACAGAAGGAGTGGCTAAGGA; Reverse prime (5’–3’): AGGCATAACGCACTAGGTTT

IL-1β; Forward primer (5’–3’): TGGTGTGTGACGTTCCC; Reverse prime (5’–3’): TGTCCATTGAGGTGGAGAG

TNF-α; Forward primer (5’–3’): GCAAAGGGAGAGTGGTCA; Reverse prime (5’–3’): CTGGCTCTGTGAGGAAGG

β-actin; Forward primer (5’–3’): GGGAAATCGTGCGTGAC; Reverse prime (5’–3’): AGGCTGGAAAAGAGCCT

Gapdh; Forward primer (5’–3’): AAGAAGGTGGTGAAGCAGG; Reverse prime (5’–3’): GAAGGTGGAAGAGTGGGAGT;

### Flow cytometry and cell sorting

Whole SNpc tissues extracted from the brain were prepared as single-cell suspensions with some modifications. In brief, SNpc tissues were digested at 37 °C with DNAse I (VIC115, Vicmed) and collagenase type II (VIC080, Vicmed) in RPMI 1640 under agitation (200 rpm) for 60 min. The cells were filtered through a 100-μm cell strainer and then suspended in PBS containing 2% (wt/vol) FBS. Peripheral blood was obtained from mice by cardiac puncture, and a single-cell suspension of peripheral blood was prepared with ACK lysis buffer (KGP11100, KeyGen). After intensive washing, the cells were labeled with fluorochrome-conjugated surface marker antibodies for fluorescence-activated cell sorting (FACS) analysis. The data were analyzed with a FACSCanto II (BD Biosciences, USA), and the percentage of each cell population and mean fluorescence intensity (MFI) were analyzed using FlowJo X software (TreeStar, Inc.). Forward scatter (FSC) and side scatter (SSC) were used to gate live cells, excluding red blood cells, debris, cell aggregates, and doublets. The following antibodies were used to identify monocytes/macrophages (Mo/MΦs). In the blood, Ly6C^hi^ classical monocytes were identified as CD45^+^/CD11b^+^/Ly6G^−^/Ly6C^hi^. In the brain, infiltrating Mo/MΦs were identified as CD45^+^/CD11b^+^/Ly6G^−^/CX3CR1^+^/Ly6C^+^ [36]. The absolute count of cells was determined by flow cytometry using Counting Beads (424902, Biolegend). Ly6C-positive cells were enriched after the isolation of single cells from the blood of mice as described above [37]. The cells were stained with the fluorochrome-conjugated antibodies described above and sorted using a FACSAria Fusion cell sorter (BD Biosciences USA). The sorted cells were subsequently subjected to adoptive transfer experiments (n = 3–4 per group).

### Enzyme‑linked immunosorbent assay (ELISA)

After anesthetization, blood was collected from the right atrium, drawn into a heparinized centrifuge tube, and centrifuged at 1000× g for 20 min. The levels of soluble TREM-1 (sTREM-1), IL-6, IL-1β, and TNF-α were measured using an established ELISA kit (JL 18245, J&L Biological, China; BR6000009, BR5210104, and BR6000087, Bioleaper, China) according to the manufacturer’s instructions (n = 5 per group).

### High-performance liquid chromatography (HPLC) analysis

The levels of dopamine in the striatum were measured using an HPLC apparatus as described previously [38]. Briefly, mice were sacrificed by decapitation and the striatum was quickly removed on ice. The striatum was subsequently weighed and homogenized in perchloric acid (HClO_4_) (0.1 mol/L). After full lysis, the samples were centrifuged at 10,000 × g (4 °C) for 20 min, after which the supernatants were collected. The dopamine content in the striatum was measured using HPLC and is expressed as ng/mg equivalent of striatal tissue (n = 3–4 per group).

### Statistical analysis

All statistical analyses were performed using GraphPad Prism V 9.0. The Shapiro-Wilk test and Brown–Forsythe test were conducted for normality and variance, respectively. For the comparison between more than two groups, one-way analysis of variance (ANOVA) was used if there was a single independent variable or two-way ANOVA for two-factorial designs, followed by Bonferroni’s post hoc test to further test pairwise differences between groups. Pearson’s correlation test was applied for correlation analysis. Data are presented as the mean values for each experimental group with variation represented as ± SEM (standard error of mean). Significance levels are indicated as follows: * *P* < 0.05, ** *P* < 0.01, *** *P* < 0.001, and not significant (n.s.).

## Results

### MPTP administration causes dopaminergic neuron injury in the SNpc and motor dysfunction

**Fig. 1:**
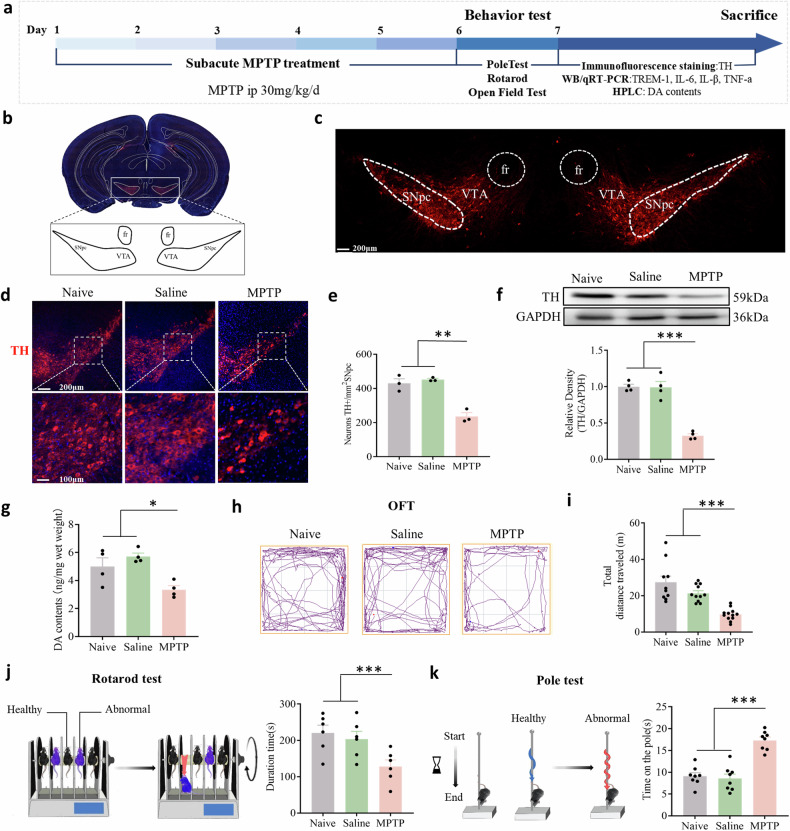
MPTP administration caused dopaminergic neuron loss in the SNpc and motor dysfunction. **a** Time course of MPTP administration and behavior tests in mice. **b**, **c** Global view of the substantia nigra in a C57BL/6 J mouse. **d**, **e** Representative images of immunofluorescence staining of TH and quantification of TH^+^ dopaminergic neurons in the SNpc. Scale bars: 200 μm for the top row and 100 μm for the bottom row. (n = 9 sections/3 mice per group). **f** Western blot analysis of TH in the SNpc of naive mice and mice treated with saline or MPTP (n = 4). **g** MPTP injection significantly decreased striatal dopamine content as measured by HPLC (n = 4). **h** Movement paths in OFT in different experimental groups. **i** Total distance moved in the OFT (n = 8–11). **j** Latency to fall off the rod on the rotarod (n = 6). **k** Latency to descend in the pole (n = 8). The data are presented as the mean ± SEM. (*P < 0.05, **P < 0.01, or ***P < 0.001 by one-way ANOVA).

We established a subacute PD mouse model by intraperitoneal injection of MPTP (30 mg/kg for 5 consecutive days), and a series of behavioral assessments such as OFT, rotarod test, and pole test were performed on Day 1 after the last MPTP injection to detect motor dysfunction (Fig. 1a). The substantia nigra is located in the midbrain posterior to the cerebral peduncle and is divided into the SNpc and the substantia nigra pars reticulata (SNpr). Dopaminergic neurons reside mainly in the SNpc and VTA [39] (Fig. 1b, c). Tyrosine hydroxylase is the rate-limiting enzyme in the biosynthesis of dopamine and can be defined as a marker of dopaminergic neurons. To evaluate dopaminergic neuron changes, we quantified the number of TH-positive cells and dopamine levels at 7 days after MPTP injection. Both the number of TH-positive neurons (Fig. 1d, e) and the TH protein levels (Fig. 1f) were significantly lower in the MPTP group than in the control group. Similarly, striatal dopamine levels were significantly lower in MPTP-injected mice than in control (N + Sal) mice (Fig. 1g), as measured via HPLC. Mice injected with MPTP exhibited decreased locomotor activity. In contrast to those in the naive and saline groups, the MPTP-injected mice traveled shorter distances in the OFT (Fig. 1h, i) and had a significantly shorter latency to fall in the rotarod test (Fig. 1j). In the pole test, MPTP injection resulted in an increase in the time taken to reach the bottom (Fig. 1k). These results indicated that the MPTP-induced PD model was successfully established in mice.

### Monocytes are needed for dopaminergic neuron and behavioral deficits in PD model mice

**Fig. 2:**
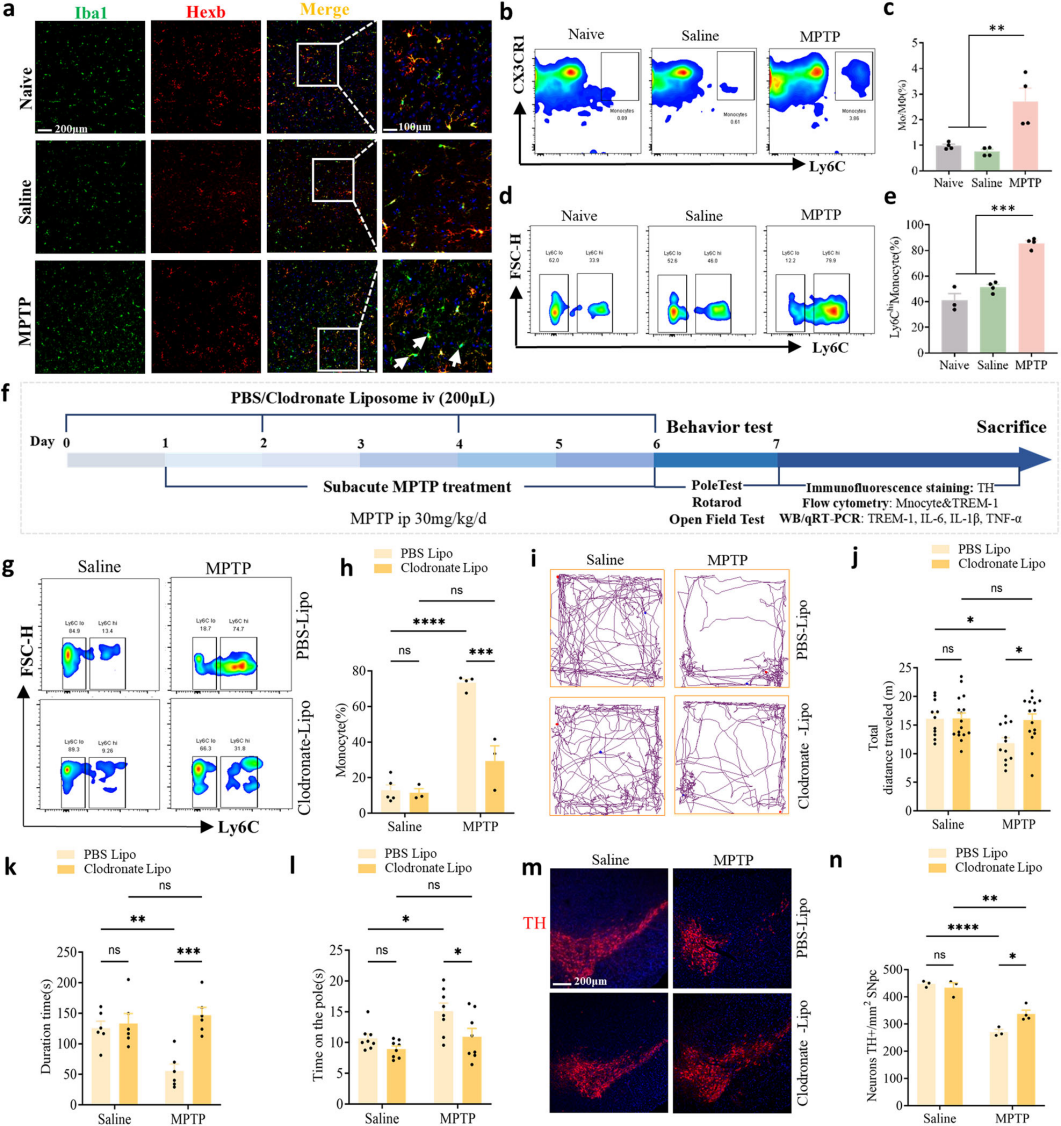
Mo/MΦs infiltrated the SNpc of PD model mice. **a** Mo/MΦs infiltration in the SNpc was demonstrated by staining for Hexb and Iba1; infiltrating Mo/MΦs are Iba1^+^/Hexb^−^ cells, and microglia are Iba1^+^/Hexb^+^ cells. Scale bars: 200 µm for the overview (left) and 100 µm for the detail (right). **b** Plots showing Mo/MΦs in the SNpc. **c** Percentages of Mo/MΦs detected in the SNpc by flow cytometry. **d** Plots showing Ly6C^hi^ monocytes in peripheral blood. **e** Percentages of Ly6C^hi^ monocytes detected in peripheral blood by flow cytometry. **f** Schematic representation of the CLP intervention therapy. Mice were intravenously injected with PBS liposomes (200 µL) or CLP (200 µL) three times 2 days apart. **g** Plots showing Ly6C^hi^ monocytes in peripheral blood. **h** Percentages of LY6C^hi^ monocytes detected in peripheral blood by flow cytometry (n = 3–5). **i** Movement paths in OFT in different experimental groups. **j** Total distance moved in the OFT (n = 11–15). **k** Latency to fall off the rod in the rotarod test (n = 6–8). **l** Latency to descend in the pole (n = 6–7). **m**, **n** Representative photographs of immunofluorescent staining of TH and quantification of the total number of TH^+^ dopaminergic neurons in the SNpc (n = 9–12 sections/3–4 mice per group). Scale bars: 200 µm for the overview. The data are presented as mean ± SEM. (*P < 0.05, **P < 0.01, or ***P < 0.001 by two-way ANOVA).

We next investigated whether the dopaminergic neuron injury in the SNpc and motor dysfunction in PD model mice require the involvement of monocytes. Monocytes are a subset of myeloid cells that play critical roles in the peripheral immune system [13]. Under several disease conditions, monocytes can infiltrate the brain parenchyma [16]. To evaluate the activation status of innate immune cells in PD model mice, we first examined the resident and infiltrating Mo/MΦs in the SNpc. Recent massive single-cell analyses revealed that Hexb is exclusively expressed in brain microglia but not in Mo/MΦs [37, 40]. Importantly, this newly defined microglia-specific gene identified Hexb as a stably expressed microglial core gene during homeostasis and disease, Iba1 is a calcium-binding protein both expressed in microglia and Mo/MΦs. Double-staining allows visualization of infiltrating Mo/MΦs in the brain based on their expression of Iba1 and lack of colocalization with Hexb, green fluorescence indicates infiltrating Mo/MΦs (white arrow). Our results revealed that infiltrating Mo/MΦs were present in the SNpc of PD model mice (Fig. 2a). Flow cytometry was used to determine the proportions and numbers of infiltrating Mo/MΦs in the SNpc of these mice. In these studies, we used CD45, CD11b, CX3CR1, Ly6C, and Ly6G as markers to reliably discriminate microglia (CD45^+^/CD11b^+^/Ly6G^−^/CX3CR1^+^) from infiltrating monocytes (CD45^+^/CD11b^+^/Ly6G^−^/CX3CR1^+^/Ly6C^+^). Full gating strategies from representative plots are shown in Supplementary Fig. S1 gating strategy.

We found that both the proportion and number of Ly6C^+^ Mo/MΦs were increased in the SNpc (Fig. 2b, c) and that Ly6C^hi^ monocytes were also detected at a greater frequency in the peripheral blood of PD model mice than in that of control mice (Fig. 2d, e; Supplementary Fig. S2). To determine whether peripheral monocytes result in dopaminergic neuron and behavioral deficits in PD model mice, we performed in vivo monocyte depletion using CLP (Fig. 2f). Flow cytometry analysis revealed a marked decrease in proinflammatory monocytes in the peripheral blood of the PD model mice that received CLP (Fig. 2g, h). The nondepleted control group received clodronate or PBS injection, which did not cause apparent infection or motor deficit. We found that saline-injected mice that received PBS liposomes or CLP behaved normally. However, compared with PD model mice, PD model mice that received CLP traveled more of a distance than PD model mice that received PBS liposomes (Fig. 2i, j); moreover, compared with PD model mice that received CLP, PD model mice that received PBS liposomes had a significantly decreased latency to fall in the rotarod test (Fig. 2k). Similarly, the PD model mice that underwent CLP exhibited a decrease in the time taken to reach the bottom in the pole test (Fig. 2l). Consistent with these changes in behaviors, the PD model mice received CLP presented a greater number of dopaminergic neurons than the PD model mice (Fig. 2m, n). In addition, we measured TH expression and dopamine levels in the striatum. The results showed that PD model mice treated with CLP had significantly higher TH expression and dopamine levels compared to those receiving PBS liposomes (Supplementary Fig. S3), further supporting the protective effect of clodronate on dopaminergic neurons. MPTP toxicity depends on the enzymatic conversion of MPTP to MPP^+^ by monoamine oxidase. To exclude the possibility that the administration of CLP affects MPTP metabolism, we measured striatal MPP^+^ levels 90 min after MPTP application. Similar levels of MPP^+^ were observed in PBS liposome-treated mice and CLP-treated mice, indicating that MPTP metabolism was not influenced by CLP treatment (Supplementary Fig. S4). These results indicated that peripheral monocytes mediate dopaminergic neuron loss and motor dysfunction in PD model mice.

### TREM-1 was elevated in peripheral infiltrating monocytes in the SNpc

**Fig. 3:**
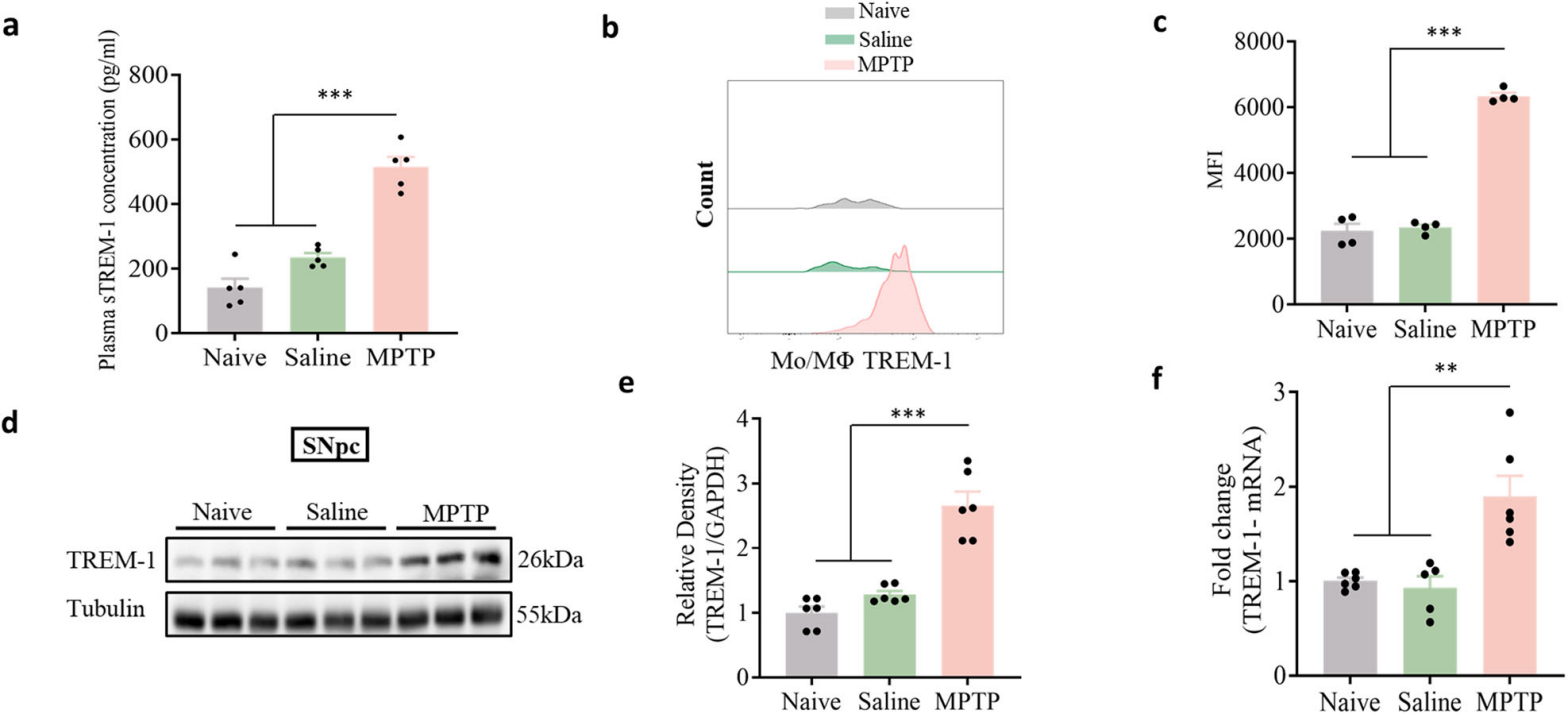
The expression of TREM-1 is upregulated in PD model mice. **a** The plasma level of sTREM-1 was significantly increased (n = 5). **b**, **c** Representative histograms of monocyte TREM-1 expression and the monocyte TREM-1 MFI in the SNpc. **d**, **e** Western blot analysis of TREM-1 in the SNpc of naive mice and mice treated with saline or MPTP (n = 6). **f** The expression level of TREM-1 in the SNpc was analyzed via qRT-PCR (n = 6). The data are presented as the mean ± SEM. (*P < 0.05, **P < 0.01, or ***P < 0.001 by one-way ANOVA).

To investigate whether TREM-1 contributes to the progression of PD, we detected the expression of TREM-1 in the SNpc. We found that sTREM-1 in the plasma was significantly increased (Fig. 3a). Flow cytometry revealed a marked increase in the TREM-1 MFI in infiltrating CD45^+^/CD11b^+^/Ly6C^+^ Mo/MΦs in the SNpc of PD model mice (Fig. 3b, c). Consistent with these data, WB and qRT-PCR analyses indicated that the expression of TREM-1 was significantly greater in PD model mice than in control mice (Fig. 3d–f). These results revealed the possible involvement of TREM-1 in PD pathogenesis. Regulating the peripheral immune response with agents that target TREM-1 may be useful for improving PD progression.

### Monocytes contribute to the increase in TREM-1 and proinflammatory cytokine levels in the SNpc

**Fig. 4:**
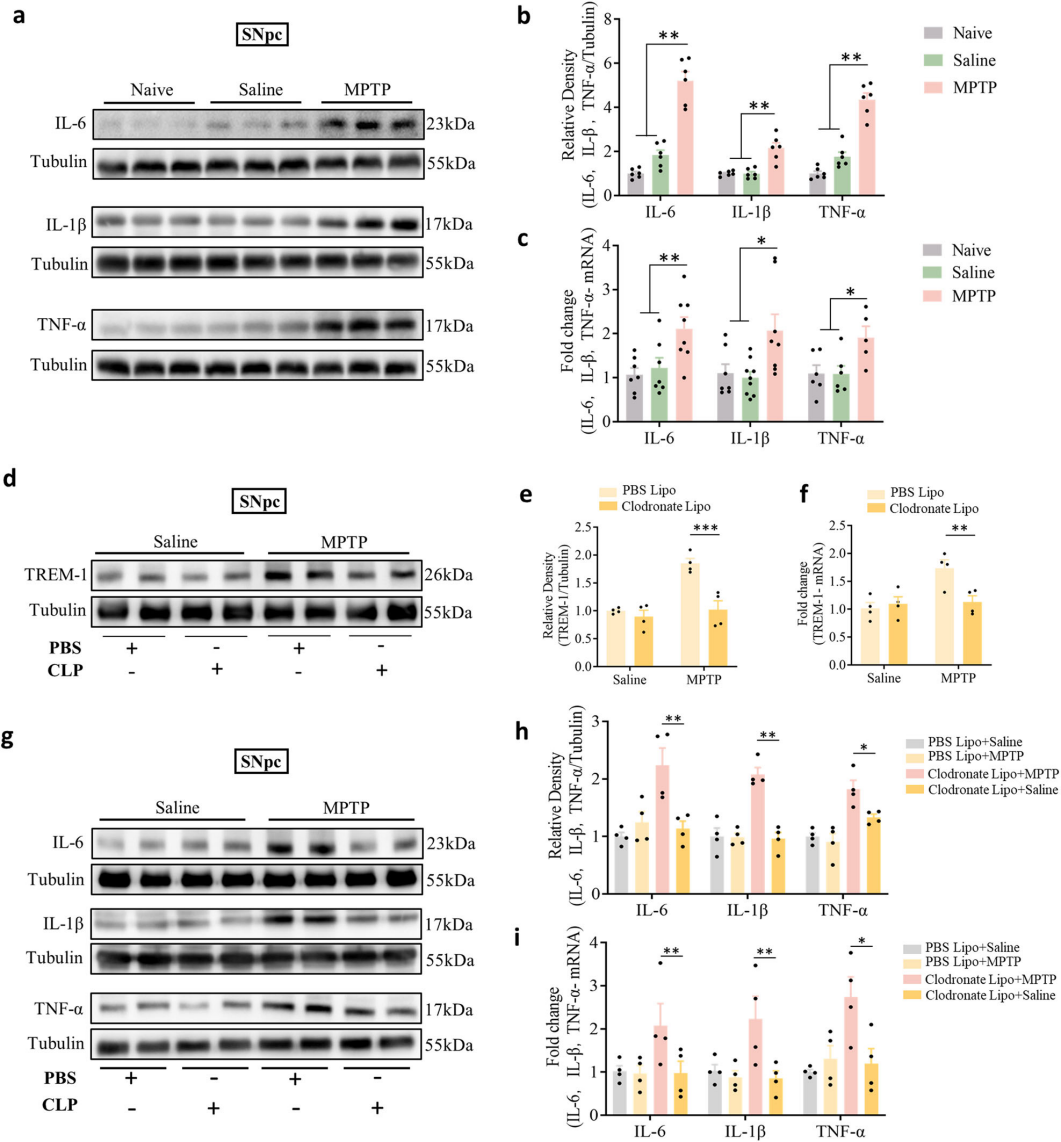
Monocyte depletion decreases TREM-1 levels in the SNpc and neuroinflammation. **a**, **b** Western blot analysis of IL-6, IL-1β, and TNF-α in the SNpc of naive mice and mice treated with saline or MPTP (n = 6). **c** The expression levels of IL-6, IL-1β, and TNF-α in the SNpc were analyzed via qRT-PCR (n = 6–8). **d**, **e** Western blot analysis of TREM-1 in the SNpc of MPTP-injected mice that underwent CLP or PBS (n = 6). **f** The expression level of TREM-1 in the SNpc was analyzed via qRT-PCR after monocyte depletion (n = 4). **g**, **h** Western blot analysis of IL-6, IL-1β, and TNF-α in the SNpc of MPTP-injected mice that underwent CLP or PBS (n = 4). **i** The expression levels of IL-6, IL-1β, and TNF-α in the SNpc were analyzed via qRT-PCR after the depletion of monocytes (n = 4). The data are presented as the mean ± SEM. (*P < 0.05, **P < 0.01, or ***P < 0.001 by one-way ANOVA and two-way ANOVA).

These results suggest that peripheral blood monocytes are critical for MPTP-induced dopaminergic neuron and motor deficits. To explore the molecular mechanisms mediated by monocytes, we first measured the protein levels of proinflammatory cytokines in the SNpc of PD model mice. WB and qRT-PCR analyses confirmed that, compared with those in the naive and saline groups, the PD model mice exhibited significantly greater IL-6, IL-1β, and TNF-α concentrations (Fig. 4a–c). The correlation analysis showed a significant correlation between the inflammatory cytokines (IL-1β, IL-6, and TNF-α) in the SNpc and motor deficit (Supplementary Fig. S5). To determine whether monocytes are needed for the MPTP-induced increase in TREM-1 levels in the SNpc, we performed WB analysis in mice that were depleted of monocytes. Our study revealed significantly lower amounts of TREM-1 (Fig. 4d–f) and proinflammatory cytokines in PD model mice depleted of monocytes by CLP than in mice with intact peripheral immune cells (Fig. 4g–i). These results suggested that monocytes are needed to increase TREM-1 levels in the SNpc and amplify neuroinflammation in PD model mice.

### TREM-1 knockout alleviates neuroinflammation, dopaminergic neuron injury, and motor dysfunction in PD model mice

**Fig. 5:**
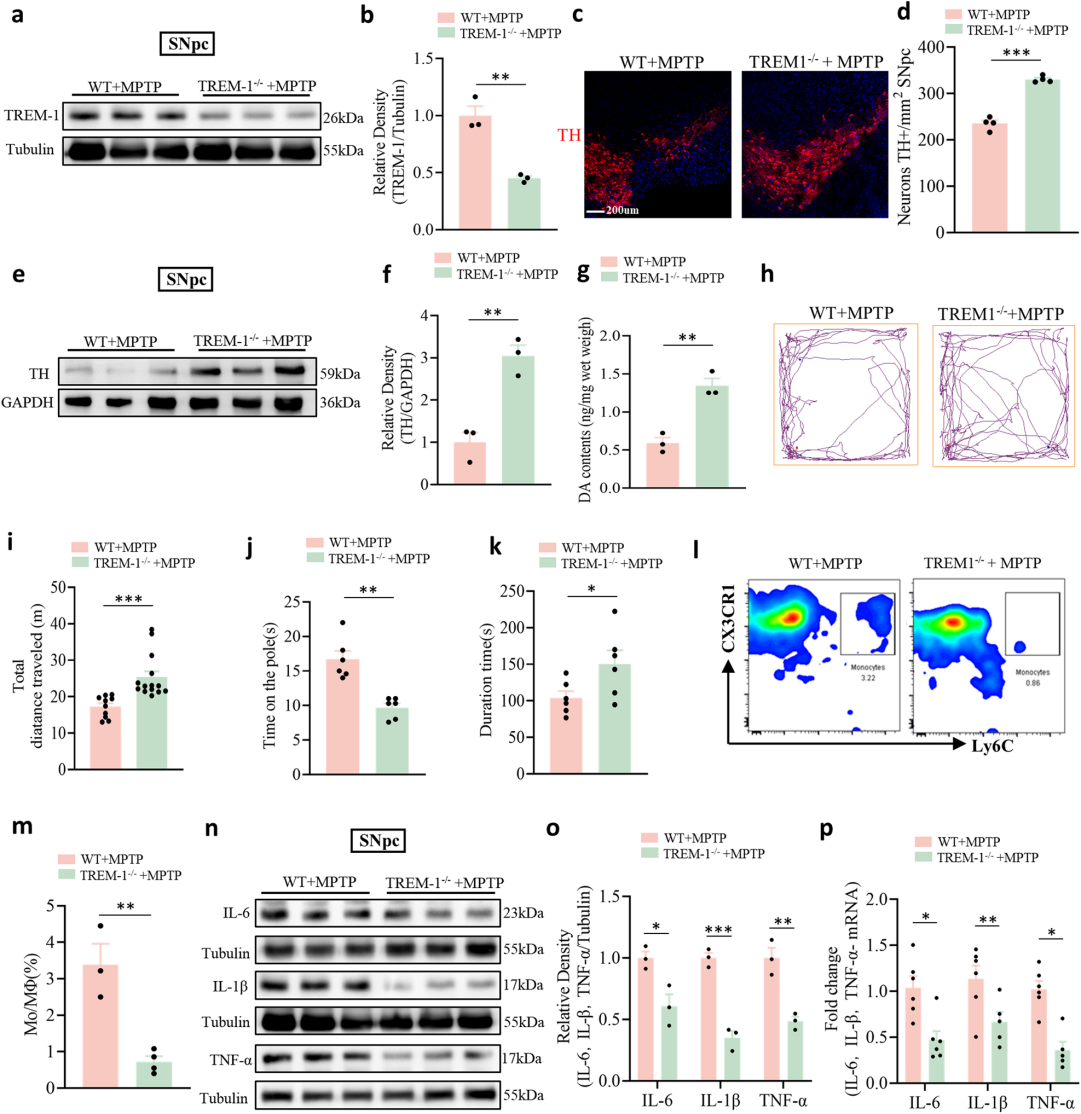
Changes induced by TREM-1 deficiency in PD model mice. **a**, **b** Western blot analysis of TREM-1 in the SNpc of WT and TREM-1-deficient mice treated with MPTP (n = 3). **c**, **d** Quantification of the total number of TH^+^ cells in the SNpc (n = 12 sections/4 mice per group). Scale bars: 200 µm for the overview. **e**, **f** Western blot analysis of TH in the SNpc of WT and TREM-1-deficient mice treated with MPTP (n = 3). **g** TREM-1 gene knockout significantly increased the striatal dopamine concentration, as measured by HPLC (n = 3). **h** Movement paths in OFT in different experimental groups. **i** Total distance moved in the OFT (n = 11–14). **j** Latency to descend in the pole (n = 6). **k** Latency to fall off the rod in the rotarod test (n = 6). **l** Plots showing Mo/MΦs in the SNpc. **m** Percentages of Mo/MΦs detected in the SNpc by flow cytometry (n = 4). **n**, **o** Western blot analysis of IL-6, IL-1β, and TNF-α in the SNpc of WT and TREM-1-deficient mice treated with MPTP (n = 3). **p** The expression levels of IL-6, IL-1β, and TNF-α in the SNpc were analyzed via qRT-PCR (n = 6). The data are presented as the mean ± SEM. (*P < 0.05, **P < 0.01, or ***P < 0.001 by Student’s t test).

We next tested the contribution of TREM-1 expressed on myeloid cells in general to PD incidence. We took advantage of mice deficient in TREM-1 in the myeloid lineage (Fig. 5a, b). Five days after the first MPTP injection, the remaining TH-positive dopaminergic neurons in the SNpc were assessed by immunofluorescence. Our data show that TREM-1 knockout did not affect dopamine neurons and inflammatory cytokines in normal mice. Furthermore, TREM-1 deficiency had no impact on motor function or emotion-related behaviors, including depression-like behavior (Supplementary Fig. S6). In MPTP-treated mice, a significant depletion of TH-positive neurons was observed in the SNpc, whereas *Trem-1*^−^^*/*^^−^ mice displayed preserved TH-positive neuron populations, indicating a pronounced resistance to MPTP-induced neurodegeneration (Fig. 5c, d). By knocking out the TREM-1 gene we observed a significant increase in TH levels in SNpc (Fig. 5e, f) and striatal dopamine levels (Fig. 5g), this alteration notably alleviated motor dysfunction in PD model mice, as evidenced by their improved performance on behavioral tests including the OFT, pole test, and rotarod test (Fig. 5h–k). Flow cytometry analysis revealed a significant decrease in the infiltration of Mo/MΦs in the SNpc of *Trem-1*^−^^*/*^^−^ mice injected with MPTP (Fig. 5l, m; Supplementary Fig. S7a). These results indicated that TREM-1 mediated the infiltration of peripheral circulating monocytes in the SNpc. We assessed the effect of genetic ablation of TREM-1 on inflammatory cytokine expression. Compared with those in *Trem-1*^−^^*/*^^−^ mice, the release of the proinflammatory cytokines IL-6, IL-1β, and TNF-α in the SNpc was markedly greater in MPTP-treated mice (Fig. 5n–p).

### Pharmacological neutralization of TREM-1 reduces the production of inflammatory cytokines, alleviates dopaminergic neuron injury, and ameliorates motor dysfunction

**Fig. 6:**
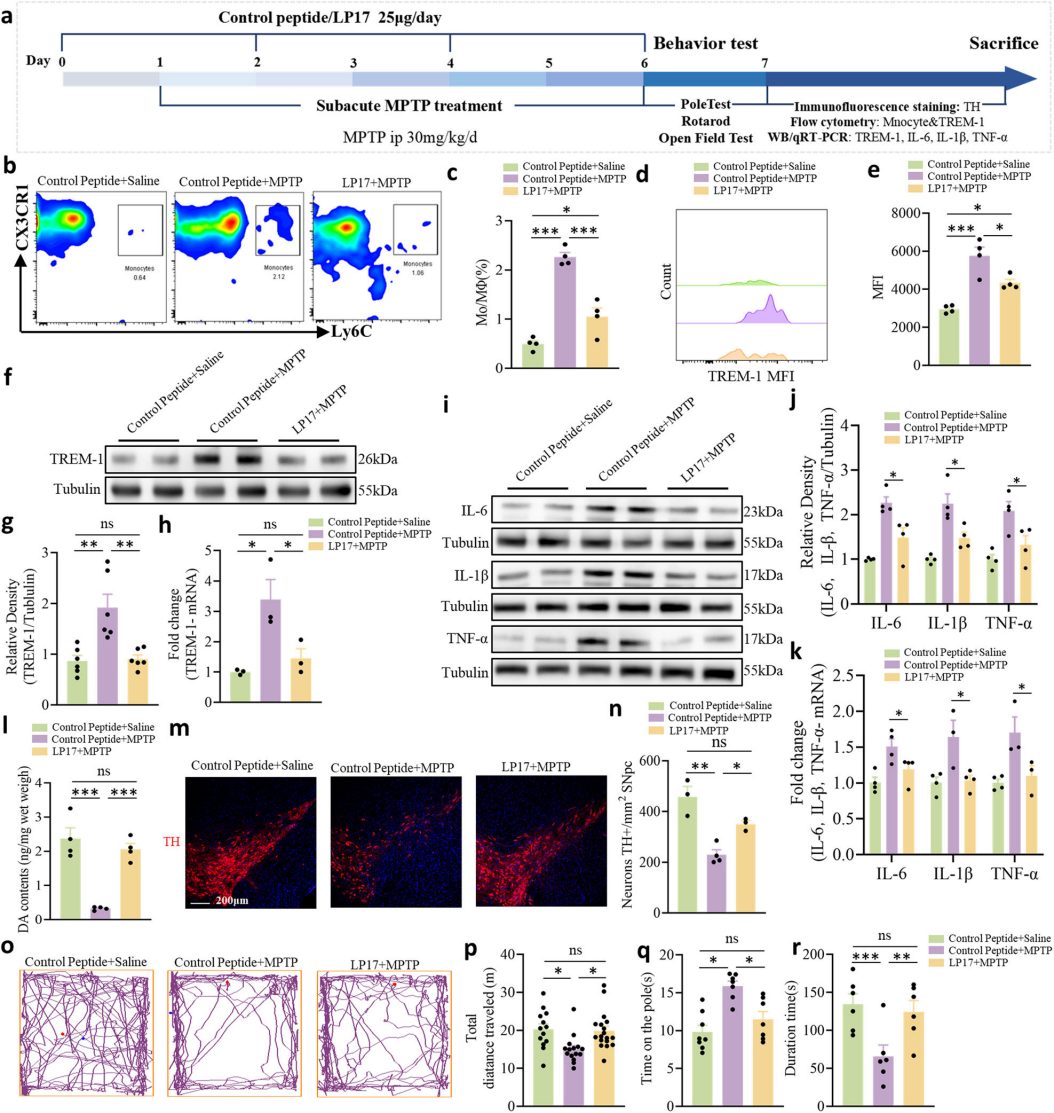
The TREM-1 decoy peptide LP17 reduces neuroinflammation and dopaminergic neuron injury. **a** Schematic representation of LP17 intervention therapy. Mice were intranasally administered four intraperitoneal injections of LP17/control peptide at 2-day intervals. **b** Plots showing Mo/MΦs in the SNpc. **c** Percentages of Mo/MΦs detected in the SNpc by flow cytometry (n = 4). **d** Representative histograms of TREM-1 expression on Mo/MΦs. **e** The MFI of Mo/MΦs TREM-1 in the SNpc (n = 4–5). **f**, **g** Western blot analysis of TREM-1 in the SNpc of MPTP-injected mice treated with LP17 or control peptide compared with control mice (n = 3). **h** The expression levels of TREM-1 in the SNpc were analyzed via qRT-PCR (n = 3). **i**, **j** Western blot analysis of IL-6, IL-1β, and TNF-α in the SNpc of MPTP-injected mice treated with LP17 or control peptide compared with control mice (n = 4). **k** The expression levels of IL-6, IL-1β, and TNF-α in the SNpc were analyzed via qRT-PCR (n = 3–4). **l** Pharmacological inhibition of TREM-1 significantly increased the striatal dopamine concentration, as measured by HPLC (n = 4). **m**, **n** Representative photographs of immunofluorescent staining of TH and quantification of the total number of TH^+^ dopaminergic neurons in the SNpc (n = 9–12 sections/3, 4 mice per group). Scale bars: 200 µm for the overview. **o** Movement paths in OFT in different experimental groups. **p** Total distance moved in the OFT (n = 12–16). **q** Latency to descend in the pole (n = 9–10). **r** Latency to fall off the rod in the rotarod test (n = 7–8). The data are presented as the mean ± SEM. (*P < 0.05, **P < 0.01, or ***P < 0.001 by one-way ANOVA).

The induction of TREM-1 suggested that systemic targeting of TREM-1 might alleviate peripheral immune responses and reduce MPTP toxicity. Accordingly, we tested whether the decoy peptide LP17 (Fig. 6a), an inhibitor of TREM-1 [41], might attenuate the immune amplification of TREM-1. The LP17 blocking peptide was identified as a competitive antagonist of membrane-bound TREM-1 for its natural ligand [42]. A previous study showed that human monocytes treated with LP17 in vitro attenuated the LPS-induced induction of inflammatory cytokines, indicating the ability of LP17 to block cellular TREM-1 [43]. Further studies have suggested that in vivo treatment with LP17 improves outcomes in sepsis [43, 44], inflammatory bowel disease [45], and cancer [46]. First, we investigated the effect of LP17 on the trafficking of monocytes to the SNpc of PD model mice. We found that the administration of LP17 during MPTP injection reduced the number of brain-infiltrating Mo/MΦs (Fig. 6b, c; Supplementary Fig. S7b). The expression of TREM-1 was markedly decreased in LP17-treated mice (Fig. 6d–h). Consistent with the results above, inflammatory cytokine expression was detected via WB and qRT-PCR in all the groups, and LP17-treated mice partially prevented the MPTP-induced release of IL-1β, IL-6, and TNF-α (Fig. 6i–k). Our study revealed that LP17 treatment also increased striatal dopamine levels (Fig. 6l) and attenuated MPTP-induced dopaminergic neuron loss (Fig. 6m, n) and motor dysfunction (Fig. 6o–r). After the pharmacologic blockade of TREM-1 with the synthetic peptide LP17, the inflammatory response in the SNpc and dopaminergic neuron injury was substantially alleviated. LP17 was similarly intranasally administered as described previously [32]. LP17 was labeled with rhodamine according to previous methods [32]. We showed that intranasally injected LP17 could penetrate the brain (Supplementary Fig. S8a). In addition, LP17 levels were determined by HPLC from SNpc (Supplementary Fig. S8b). We believe the LP17 reached an effective therapeutic concentration in the brain and the expression of TREM-1 was effectively inhibited. Intranasal drug administration is an efficient and noninvasive method for bypassing the BBB and rapidly targeting various chemicals or peptides to the brain, which is valuable for clinical translation [47]. To exclude the possibility that the protective effect of LP17 was due to alterations in MPTP metabolism, we measured the MPP^+^ concentration via HPLC and found that intranasal administration of LP17 did not affect the concentration of MPP^+^, the metabolite of MPTP, in the striatum (Supplementary Fig. S9).

### Infiltrating peripheral monocyte TREM-1 mediates dopaminergic neuron injury and neuroinflammation in PD model mice

Given that monocytes are needed for dopaminergic neuron injury, motor dysfunction, and elevated TREM-1 levels in the SNpc, the pathogenesis of PD model mice is likely mediated by peripheral monocytes through TREM-1 signaling. Therefore, we next explored whether monocytes induce dopaminergic neuron injury and motor deficits in a TREM-1-dependent manner.

**Fig. 7:**
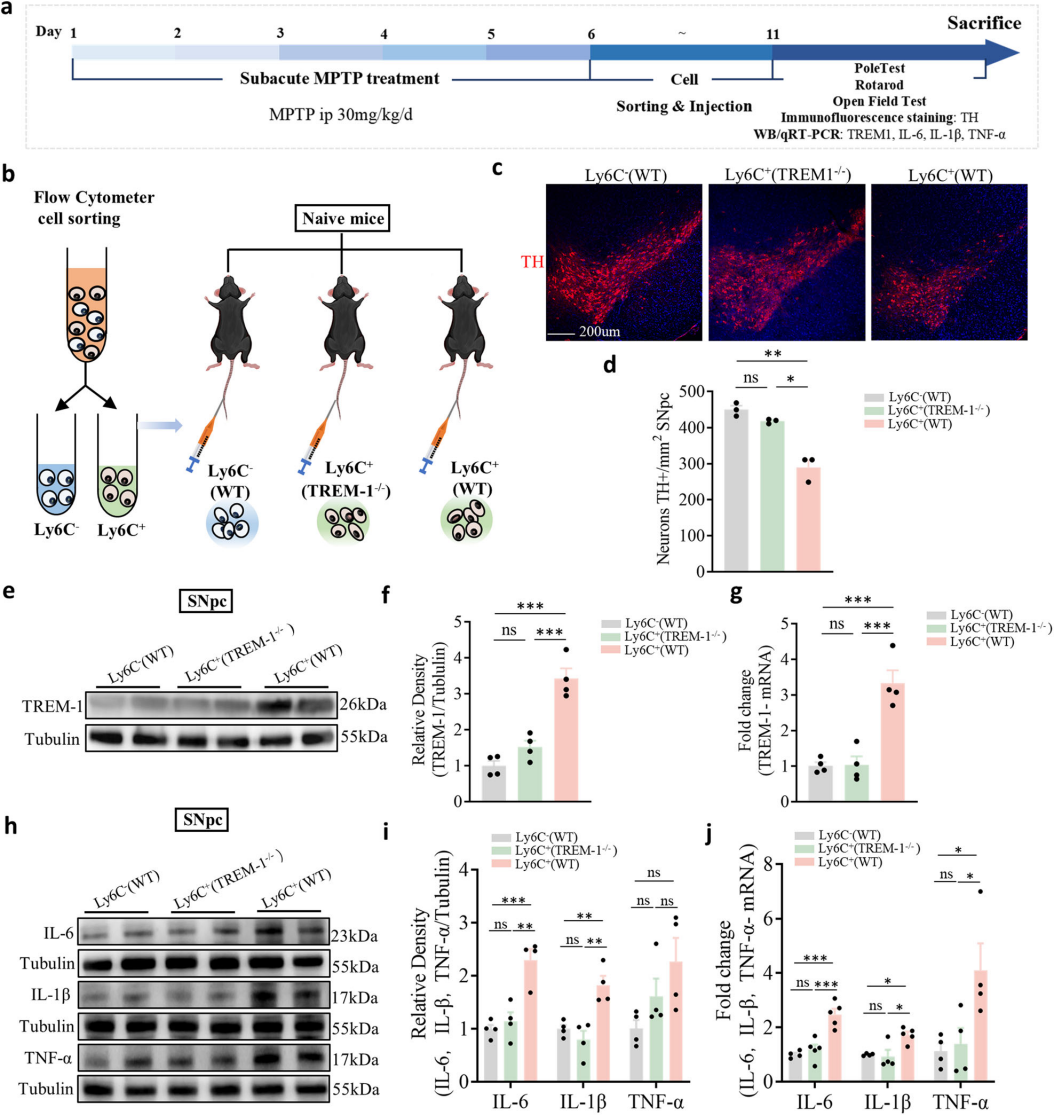
Administration of TREM-1-producing monocytes sorted from PD model mice induces dopaminergic neuron injury and neuroinflammation in naive mice. **a** Experimental design. **b** Ly6C^+^ cells were collected from the peripheral blood of WT or *Trem-1*^*−/−*^ donor mice 24 h after MPTP injection and sorted based on the surface expression of Ly6C. Naive mice were injected with 3 × 10^6^ Ly6C^+^ or Ly6C^−^ cells on Day 11. **c**, **d** Quantification of the total number of TH^+^ dopaminergic neurons in the SNpc (n = 9 sections/3 mice per group). Scale bars: 200 µm for the overview. **e**, **f** Western blot analysis of TREM-1 in the SNpc of recipient mice that were injected with Ly6C^+^ or Ly6C^−^ cells from WT MPTP-injected mice or *Trem-1*^*−/−*^ MPTP-injected mice (n = 4). **g** Expression levels of TREM-1 in the SNpc determined by qRT-PCR (n = 4). **h**, **i** Western blot analysis for IL-6, IL-1β, and TNF-α in the SNpc of recipient mice that were injected with Ly6C^+^ or Ly6C^−^ cells from WT MPTP-injected mice or *Trem-1*^*−/−*^ MPTP-injected mice (n = 4). **j** The expression levels of IL-6, IL-1β, and TNF-α in the SNpc were analyzed via qRT-PCR (n = 4). The data are presented as the mean ± SEM. (*P < 0.05, **P < 0.01, or ***P < 0.001 by one-way ANOVA).

In this study, we collected monocytes from the peripheral blood of WT and *Trem-1*^−^^*/*^^−^ mice after MPTP injection and sorted the cells based on the surface expression of CD45, CD11b and Ly6C by FACS (Fig. 7a). The naive mice were intravenously injected with 3 × 10^6^ sorted CD45^+^/CD11b^+^/Ly6C^+^ and CD45^+^/CD11b^+^/Ly6C^−^ cells (Fig. 7b). Five days after sorted cell transfer, we observed a substantial decrease in the number of dopaminergic neurons in naive mice that received Ly6C^+^ cells from PD model mice compared to that in naive mice that received Ly6C^−^ cells from PD model mice. Notably, there were no differences in the number of dopaminergic neurons in naive mice that received Ly6C^+^ cells collected from *Trem-1*^*−/−*^ PD model mice (Fig. 7c, d). In addition to dopaminergic neuron loss, we conducted behavioral assessments to evaluate motor function in naive mice that received Ly6C^+^ cells. As shown in Supplementary Fig. S10, naive mice injected with Ly6C^+^ cells from PD model mice exhibited significant motor deficits in both the open field and rotarod tests, indicating impaired motor coordination and mobility. Although there was a trend toward reduced performance in the pole test, this difference did not reach statistical significance. These findings suggest that Ly6C^+^ cell transfer leads to partial motor dysfunction, which correlates with dopaminergic neuron loss.

We also found that the expression of TREM‐1 was significantly increased in the naive mice that received Ly6C^+^ cells from PD model mice (Fig. 7e–g). We subsequently detected inflammatory cytokine expression in the SNpc and serum, and compared with naive mice injected with Ly6C^−^ cells, naive mice injected with Ly6C^+^ cells exhibited markedly increased levels of IL-6, IL-1β, and TNF-α (Fig. 7h–j, Supplementary Fig. S11). These results support the theory that TREM-1 is an inflammatory response amplifier and suggest that the administration of TREM-1-producing monocytes alone is sufficient to induce both motor dysfunction and dopaminergic neuron loss in PD model mice, as well as to trigger neuroinflammation.

## Discussion

Accumulating evidence highlights the involvement of innate immune cells in PD, but the neuroimmune mechanisms underlying the infiltration of monocytes into the SNpc in PD remain unclear. Taken together, the results of our study indicate that the amplification of peripheral monocytes by TREM-1 is involved in the aggravation of dopaminergic neurodegeneration in MPTP-induced PD model mice. Genetic ablation of TREM-1 or LP17 blockade prevented the loss of dopaminergic neurons in the SNpc. We presume this effect was predominantly based on the inhibition of the TREM-1-mediated peripheral innate immune response and brain-infiltrating monocytes. These dopaminergic neuron and behavioral deficits were prevented by in vivo deletion of peripheral monocytes or ablation of TREM-1, both of which attenuated the increase in TREM-1 signaling in response to MPTP. Together, our findings reveal the underlying mechanism of neuroinflammation in PD and highlight monocyte TREM-1 signaling as a potential target for attenuating the neurodegeneration effects of PD.

A previous study revealed that the inflammatory component of PD was driven by myeloid cells, including resident microglia and infiltrating peripheral Mo/MΦs [48]. Clinical research has suggested that classical monocytes are enriched in the blood of PD patients [10], and our current study also demonstrated the point that PD monocytes are predisposed to inflammation, as previously mentioned [11]. These findings were confirmed by the results of a series of in vivo experiments. Initially, following the successful establishment of the PD model mice, the number of Ly6C^hi^ monocytes in the peripheral blood and infiltrating brain Mo/MΦs increased. Concurrently, the levels of inflammatory cytokines (IL-1β, IL-6, TNF-α) in the SNpc also increased. Notably, these changes showed a significant correlation with the behavioral outcomes (OFT, Pole Test, Rotarod). Moreover, the depletion of peripheral monocytes after CLP could prevent MPTP-induced loss of dopaminergic neurons and improve behavioral deficits. CLP is widely used to deplete peripheral Mo/MΦs [49–51]. These results are consistent with previous studies showing that the depletion of peripheral monocytes prevents inflammation and neurodegeneration in a model of PD [52, 53]. Additionally, while CLP is commonly regarded as a tool for the specific depletion of Mo/MΦs, emerging evidence [54] indicates that neutrophils may also be impacted by this treatment. However, in our study, flow cytometry data did not show any significant changes in neutrophil counts (Supplementary Fig. S12), suggesting that the depletion effect in this model remained primarily targeted at Mo/MΦs. Furthermore, our data confirmed that CLP treatment neither impaired nor enhanced the metabolism of MPTP to its active neurotoxic metabolite MPP+, ensuring the reliability of our model in evaluating the neuroprotective effects induced by CLP injection.

However, the specific molecular mechanisms by which monocyte TREM-1 impairs dopaminergic neurons and motor function have not been determined. In the peripheral immune system, inflammatory cytokines, which are capable of influencing the CNS, are released from the peripheral circulation to the CNS via multiple routes [55–57]. Two different forms of TREM-1 have been identified: membrane-bound TREM-1 and soluble receptor 1 (sTREM-1). Both the concentration of sTREM-1 in plasma and the expression of TREM-1 in the SNpc increase after MPTP injection, and depletion of peripheral monocytes via CLP prevents the expression of TREM-1 in the SNpc. These results suggest that peripheral TREM-1 can reach the brain parenchyma by crossing the BBB. Previous research has confirmed that MPTP-induced PD model mice exhibit increased BBB permeability [58, 59]. When peripheral monocytes TREM-1 infiltrate the brain, they can promote the release of proinflammatory cytokines including IL-6, IL-1β, and TNF-α, which ultimately leads to dopaminergic neuron damage. This study innovatively demonstrates that TREM-1 is expressed on Mo/MΦ that infiltrates the SNpc of PD model mice. As an amplifier of the inflammatory immune response, once in the brain, we speculate that TREM-1 can be sensed by microglia and that activated microglia can release additional proinflammatory cytokines; in turn, a vicious cycle is formed with persistent neuroinflammation in PD, which leads to dopaminergic neuron loss.

Available genetically modified mice have greatly advanced our understanding of the pivotal role of TREM-1 in disease. This phenomenon is best exemplified by studies on the role of TREM-1 in stroke treatment. Experiments in which mouse TREM-1 was ubiquitously ablated showed that these mice were protected against intracerebral hemorrhage-induced neurobehavioral deficits, indicating that triggering of TREM-1 on myeloid cells induces a neuroinflammatory response [60, 61]. The latest study using TREM-1 positron emission tomography tracer technology revealed infiltrating myeloid cells in the brains of PD model mice [62]. Despite the different modeling methods used, these results suggest that TREM-1 is involved in the peripheral myeloid-mediated proinflammatory innate immune response, which has implications for our study.

A novel finding of our study was that blockade of TREM-1 after MPTP injection prevents circulating peripheral monocytes from infiltrating the SNpc. Our results revealed that brain-invading Ly6C^hi^ inflammatory monocytes are drivers of neuroinflammation, dopaminergic neuron injury, and motor dysfunction, which is consistent with the role of TREM-1 in the inflammatory response [63]. Therefore, we inferred that TREM-1 plays a negative role in PD model mice. We speculate that one possibility is that global deficiency of TREM-1 prevents monocyte recruitment by quenching early neuroinflammation, namely, the production of chemokines. Another possible explanation is that TREM-1 is activated by monocytes to migrate to inflammatory sites, and inhibiting TREM-1 signaling directly on monocytes might block their recruitment to the SNpc. The current phenomenon that monocyte brain infiltration and SNpc inflammation are both inhibited by global TREM-1 depletion leads to the fascinating hypothesis that the benefits of TREM-1 antagonism might be largely attributed to blocking monocyte recruitment to the SNpc in PD model mice. Previous research has shown that the TREM-1 protein is expressed by infiltrating Mo/MΦs, not microglia, at the peak of neuroinflammation [64], reinforcing the idea that peripheral monocyte-derived TREM-1 plays a critical role in driving neuroinflammatory processes. While TREM-1 has also been observed in microglia under certain pathological conditions, such as ischemic stroke [32], our findings underscore that, in the context of Parkinson’s disease-related neuroinflammation, the predominant contribution of TREM-1 arises from infiltrating monocytes. Nonetheless, it is important to acknowledge the role of resident microglia, particularly as they may be activated further by infiltrating monocytes, thereby contributing to the overall inflammatory response (Supplementary Fig. S13).

Indeed, we have unexpectedly discovered a population of Ly6C^+^/CX3CR1^+^ monocytes that appear ungated in the TREM-1^−^^/^^−^ condition and the LP17 + MPTP condition. This distinct subset of monocytes may have varying roles in the context of the CNS, including inflammatory responses or tissue repair, depending on the environmental conditions. CX3CR1 high monocytes have been shown to infiltrate the injured tissue to differentiate into regenerative macrophages that promote neuronal protection and repair following excitotoxicity-mediated injury [65]. Our findings suggest that inhibition of TREM-1 activity reduces the inflammatory response in the brains of PD model mice, which may facilitate the differentiation of a portion of Ly6C^hi^ inflammatory monocytes transdifferentiate into CX3CR1^+^/Ly6C low ‘repair’ macrophages in the brain. We have added a supplemental figure (Supplementary Fig. S14) quantifying this population in both the TREM-1^−^^/^^−^ and LP17 + MPTP conditions. In our quantitative analysis, we found that approximately 5% of the cells under the TREM-1^−^^/^^−^ condition and 2% under the LP17 + MPTP condition are CX3CR1^+^/Ly6C low monocytes. The data shed light on the frequency of this particular cell population under the experimental conditions we studied. This hypothesis enriches our understanding of the shifting dynamics among monocyte populations in the CNS, depending on the conditions. To probe further into the role of this cell population, it might be necessary to use additional markers for a more accurate identification of these cells and a clearer delineation of their function.

Previously, Feng’s research groups utilized LP17 to knock down TREM-1 expression in a BV2 cell model and partially protect dopaminergic neurons against 6-OHDA [66]. However, our research focused on the regulatory effect of TREM-1 in the SNpc in PD patients and emphasized that TREM-1 expression on infiltrating peripheral monocytes mediates dopaminergic neuronal damage. To clarify the cell type-specific mechanisms involved, we used adoptive cell transfer in the last part of our study to test our hypothesis. Although the results of this study are quite encouraging, it still has some limitations. For adoptive transfer experiments, we primarily relied on functional markers and phenotypic characteristics to differentiate between donor and recipient cells. While the method we employed has proven its efficacy in numerous studies [67, 68], we acknowledge that it does not afford us the precision to delineate the exact proportion of circulating cells originating from the donor versus the recipient. Despite this limitation, the data and insights gleaned from our research remain valuable and informative. We will consider using the CD45.1/CD45.2 system or other methods that can more accurately label and track donor and recipient cells in future research to further enhance the accuracy and reliability of our experiments. In addition, TREM-1 is also expressed by epithelial cells, endothelial cells, lymphocytes, and platelets as previously reported [69–71]. Lymphocytes accumulate and infiltrate the CNS in PD model mice [9, 72], and we detected CD4^+^ lymphocyte infiltration into the SNpc. However, the MFI of TREM-1 expressed on CD4^+^ lymphocytes was very low and there was no significant difference between the control group and the MPTP group (Supplementary Fig. S15). Additional studies are necessary to further explore the inflammatory mechanism of PD mediated by other sources of TREM-1.

In summary, we identified TREM-1 as a key factor contributing to PD pathogenesis through the regulation of both monocyte infiltration and neuroinflammation. Targeting TREM-1 might constitute a novel and very useful therapeutic strategy to limit PD progression.

## Supplementary information

Supplementary materials

Full gel and blot images

## Data Availability

The datasets used and/or analyzed during the current study are available from the corresponding author upon reasonable request.
