# Supplementary material for: Infiltrating peripheral monocyte TREM-1 mediates dopaminergic neuron injury in substantia nigra of Parkinson’s disease model mice
Source: Cell Death Dis. 2025 Jan 14;16(1):18. doi: 10.1038/s41419-025-07333-5 (PMC11733277; doi:10.1038/s41419-025-07333-5)
Supplement: Supplementary file 1 — Supplementary materials [file 41419_2025_7333_MOESM1_ESM.pdf]

Supplementary Figures

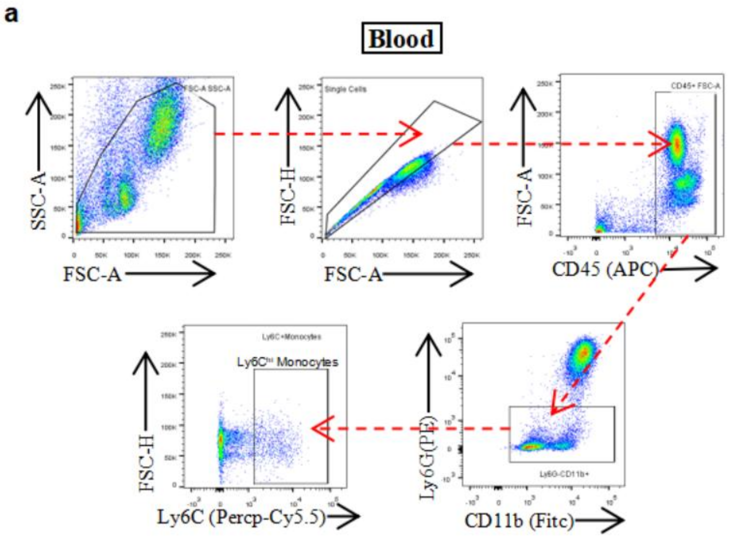

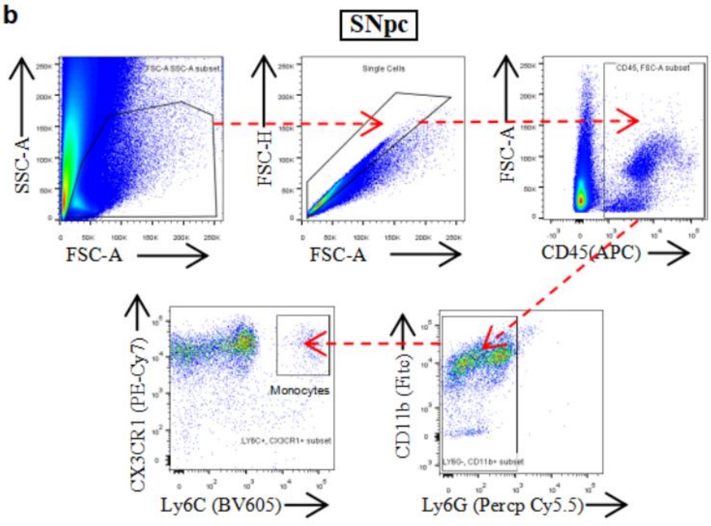

Fig. S1 Flow cytometry gating strategy and representative raw flow cytometry data in the blood (a) and in the SNpc (b).

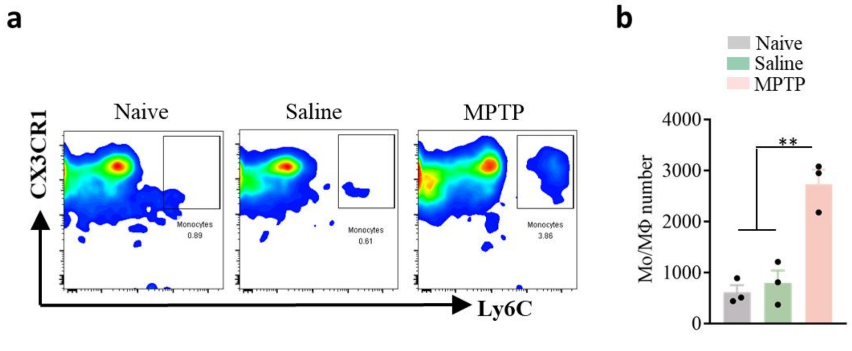

Fig. S2 Absolute number of infiltrating Mo/MΦs in the SNpc. (a) Plots showing Mo/MΦs in the SNpc. (b) The total number of CD45$^+$/CD11b$^+$/Ly6C$^+$ Mo/MΦs are increased in the SNpc after MPTP injection (n = 3). The data are presented as the mean ± SEM. (*$P < 0.05$, **$P < 0.01$, or ***$P < 0.001$ by one-way ANOVA or by Student's t-test).

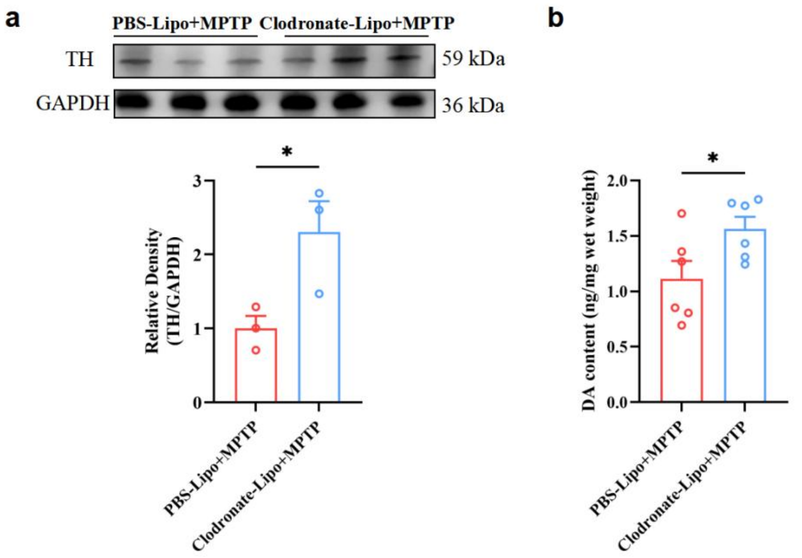

Fig. S3 Clodronate liposome treatment preserves striatal dopaminergic markers in PD model mice. (a) Western blot analysis for TH in the SNpc showed a significant increase in TH expression in CLP-treated PD model mice compared to the PBS liposome-treated group (n = 3). (b) Dopamine (DA) levels in the striatum, as measured by HPLC, were significantly higher in the CLP-treated

group (n = 3). The data are presented as the mean ± SEM. (*$P < 0.05$, **$P < 0.01$, or ***$P < 0.001$ by Student's t test).

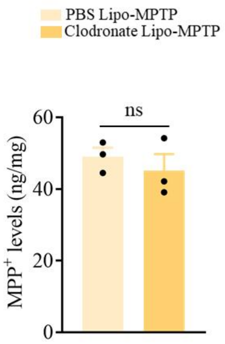

Fig. S4 MPP+ levels were determined by HPLC from the striatum dissected 90 min after intraperitoneal MPTP application. CLP application does not alter MPP+ levels striatum after MPTP injection (n = 3). The data are presented as the mean ± SEM. (*$P < 0.05$, **$P < 0.01$, or ***$P < 0.001$ by Student's t-test).

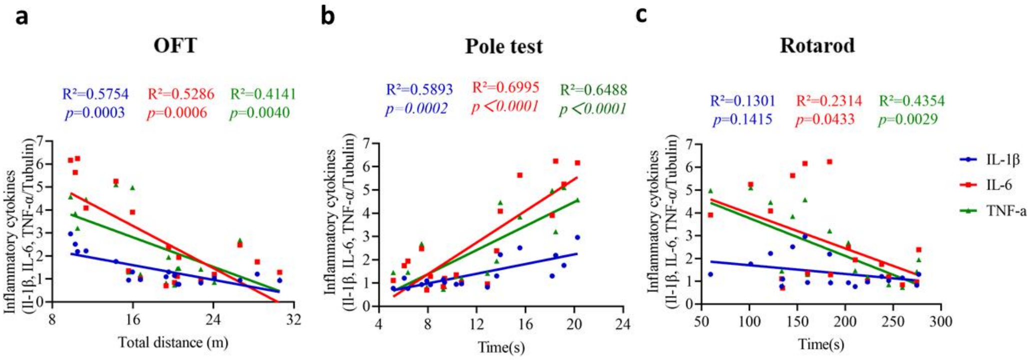

Fig. S5 Correlation analysis between the inflammatory cytokines (IL-1β, IL-6, and TNF-α) and motor deficit. (a) Total distance traveled in the OFT and inflammatory cytokines (IL-1β, IL-6, and TNF-α) level in the SNpc (n = 6). (b) Latency to fall off the rod in the rotarod and inflammatory cytokines (IL-1β, IL-6, and TNF-α) level in the SNpc (n = 6). (c) Latency to descend in the pole

and inflammatory cytokines (IL-1β, IL-6, and TNF-α) level in SNpc (n = 6). Pearson's correlation test was applied for correlation analysis. Significance levels are indicated as: * $P < 0.05$, ** $P < 0.01$, *** $P < 0.001$, and not significant (n.s.).

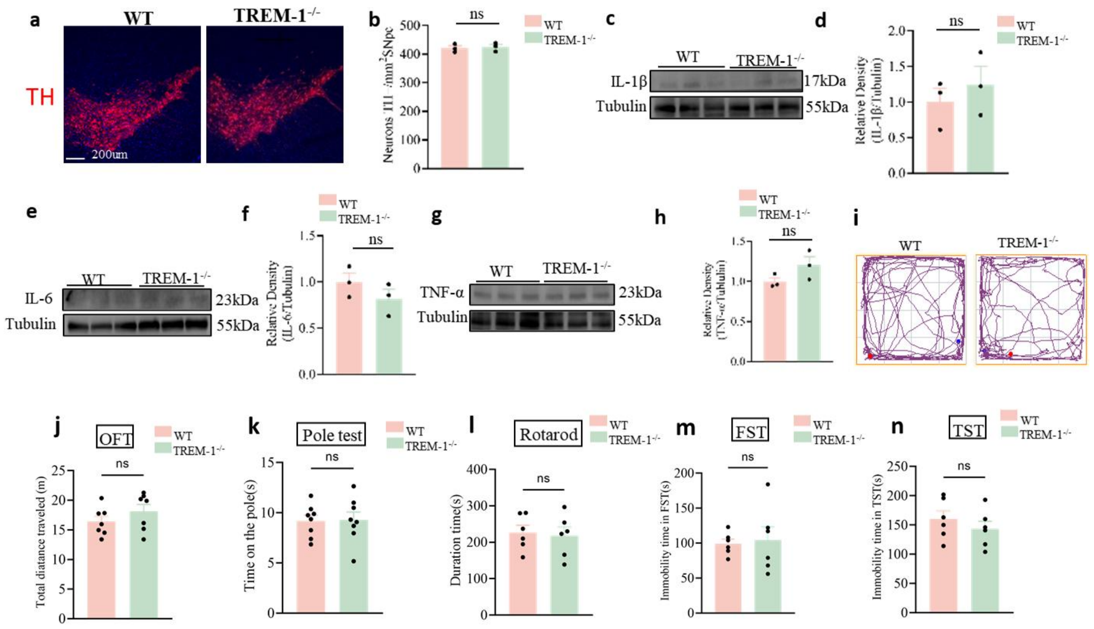

Fig. S6 Effects of TREM-1 gene knockout on the dopamine neurons, inflammatory cytokines (IL-1β, IL-6, and TNF-α), and behavioral performance. (a-b) Quantification of the total number of TH^+ cells in the SNpc (n = 6 sections/3 mice per group). Scale bars: 200 μm for the overview. (c-h) Western blot analysis for IL-1β, IL-6, and TNF-α in the SNpc of the WT and TREM-1-deficient mice (n = 3). (i) Movement paths in OFT in different experimental groups. (j) Total distance moved in the OFT (n = 7). (k) Latency to fall off the rod on the rotarod (n = 7). (l) Latency to descend in the pole (n = 7). (m) Immobility time in the forced swim test (n = 6). (n) Immobility time in the tail suspension test (n = 6). The data are presented as the mean ± SEM. (*$P < 0.05$, **$P < 0.01$, or ***$P < 0.001$ by Student's t-test)

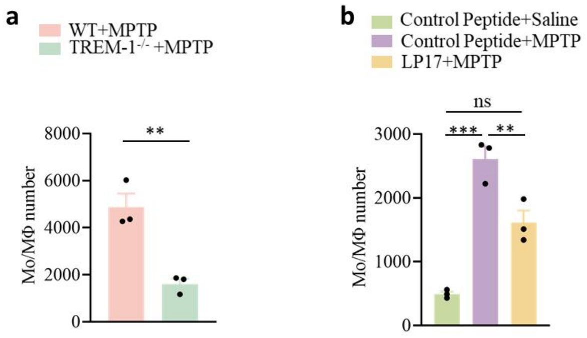

Fig. S7 Absolute number of infiltrating Mo/MΦs in the SNpc. (a) TREM-1gene knockout or (b) pharmacological inhibition significantly reduces the total number of CD45+/CD11b+/Ly6C+ Mo/MΦs in the SNpc (n = 3). The data are presented as the mean ± SEM. (**P* < 0.05, ***P* < 0.01, or ****P* < 0.001 by one-way ANOVA or by Student's t-test).

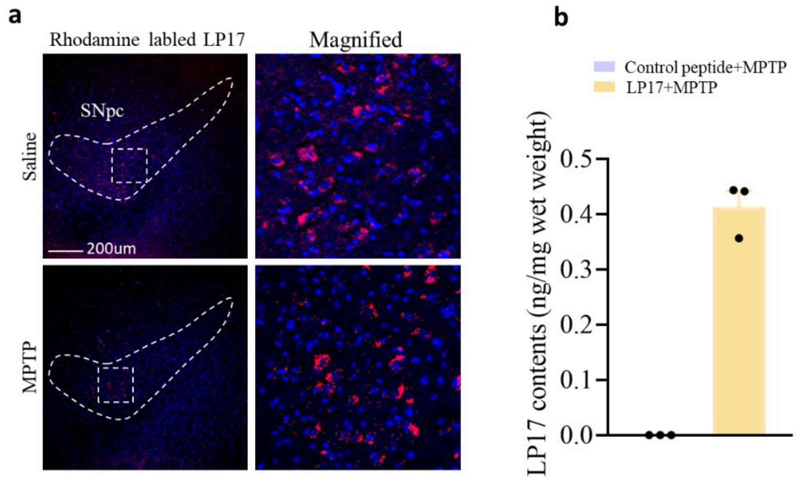

Fig. S8 LP17 could get access into the brain. (a) LP17 was labeled with rhodamine and red fluorescent signals of LP17 were detected in SNpc. (b) LP17 levels were determined by HPLC from SNpc (n = 3). The data are presented as the mean ± SEM. (**P* < 0.05, ***P* < 0.01, or ****P* < 0.001 by Student's t test).

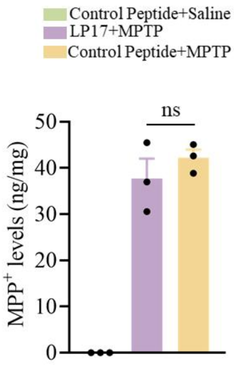

Fig. S9 MPP+ levels were determined by HPLC from the striatum dissected 90 min after intraperitoneal MPTP application. LP17 application does not alter MPP^+ levels striatum after MPTP injection (n = 3). The data are presented as the mean ± SEM. (**P* < 0.05, ***P* < 0.01, or ****P* < 0.001 by one-way ANOVA or by Student's t-test).

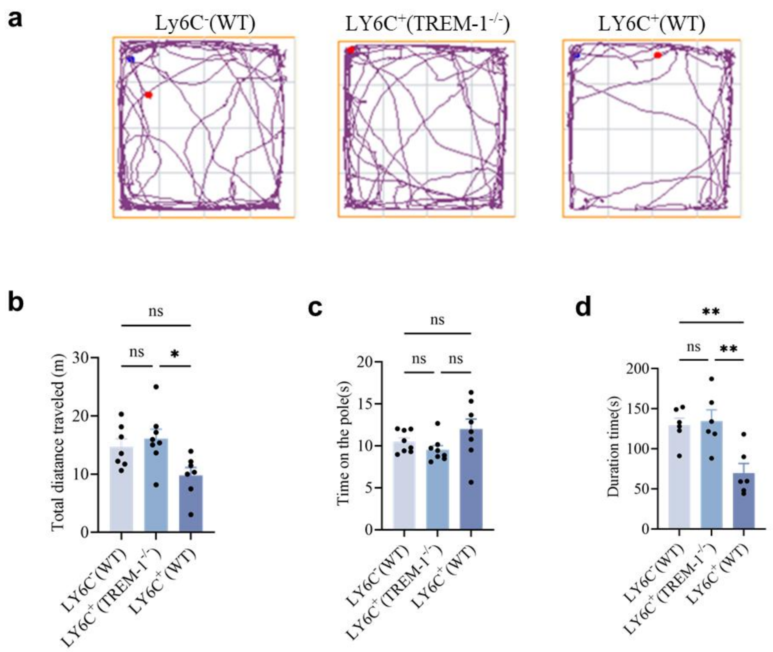

Fig. S10 Administration of TREM-1-producing monocytes sorted from PD model mice induces motor dysfunction in naive mice. (a) Movement paths in OFT in different experimental groups. (i) Total distance moved in the OFT (n = 7). (c) Latency to descend in the pole (n = 8). (d) Latency to

fall off the rod on the rotarod (n = 6). The data are presented as the mean ± SEM. (*$P < 0.05$, **$P < 0.01$, or ***$P < 0.001$ by one-way ANOVA).

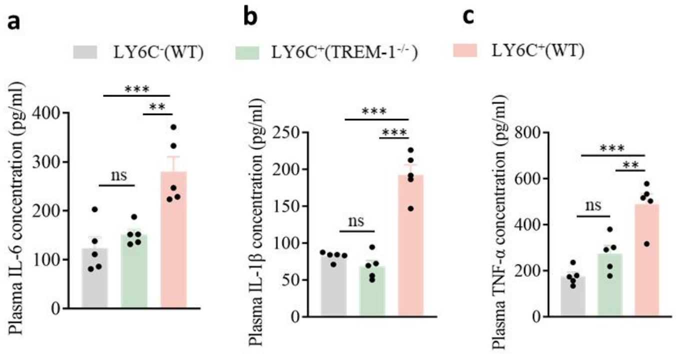

Fig. S11 Inflammatory cytokines (IL-6, IL-1β, and TNF-α) in serum after adoptive transfer of Ly6C^{+} monocytes. (a-c) IL-6, IL-1β, and TNF-α were detected in serum by ELISA (n = 5). The data are presented as the mean ± SEM. (*$P < 0.05$, **$P < 0.01$, or ***$P < 0.001$ by one-way ANOVA).

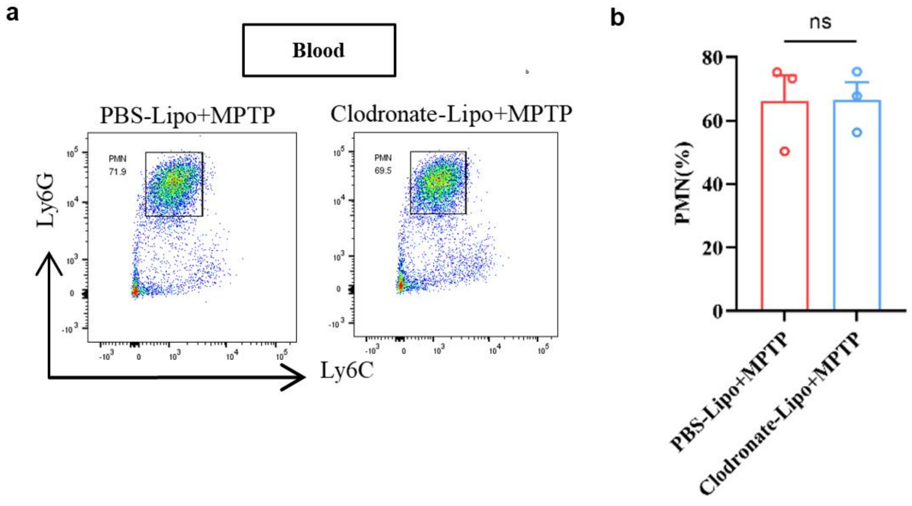

Fig. S12 Clodronate liposome treatment had no impact on neutrophil count in the PD mouse model. (l) Plots showing PMN in the blood. (m) Percentages of PMN detected in the Blood by flow cytometry (n = 3). The data are presented as the mean ± SEM. (*$P < 0.05$, **$P < 0.01$, or

***$P < 0.001$ by Student's t test).

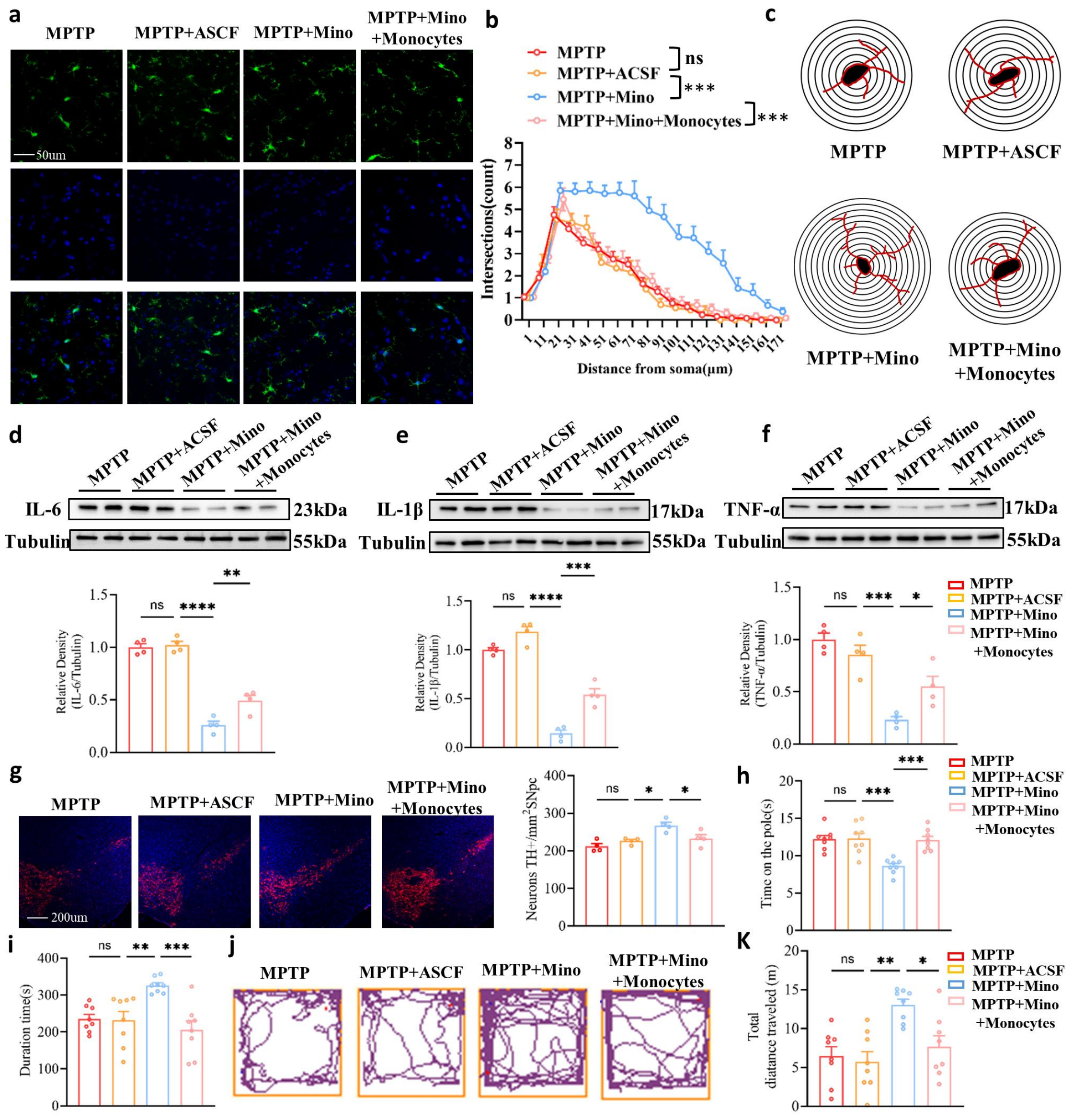

Fig. S13 Effects of microglia inhibition and monocyte transfer on on the dopamine neurons, inflammatory cytokines (IL-1β, IL-6, and TNF-α), and behavioral performance. (a-c) Sholl analysis of microglial morphology in different treatment conditions: MPTP, MPTP+ACSF, MPTP+minocycline, and MPTP+minocycline+monocytes (n = 6) Scale bars: 50 μm. (d-f) Western blot analysis of IL-6, IL-1β, and TNF-α expression in the SNpc of mice treated with MPTP, MPTP+ACSF, MPTP+minocycline, and MPTP+minocycline+monocytes (n = 4). (g) Quantification of the total number of TH^+ dopaminergic neurons in the SNpc (n = 12 sections/4 mice per group). (h) Latency to descend in the pole (n = 8). (i) Latency to fall off the rod in the

rotarod test (n = 8). (j-k) Total distance moved in the OFT (n = 8). The data are presented as the mean ± SEM. (*$P < 0.05$, **$P < 0.01$, or ***$P < 0.001$ by one-way ANOVA).

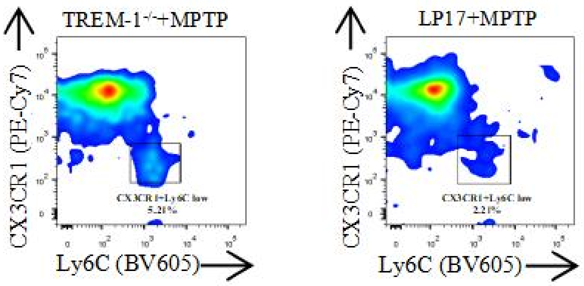

Fig. S14 Cells population of CX3CR1^{+}/Ly6C low monocytes that appear in the TREM1$^{-/-}$ + MPTP condition and in the LP17 + MPTP condition.

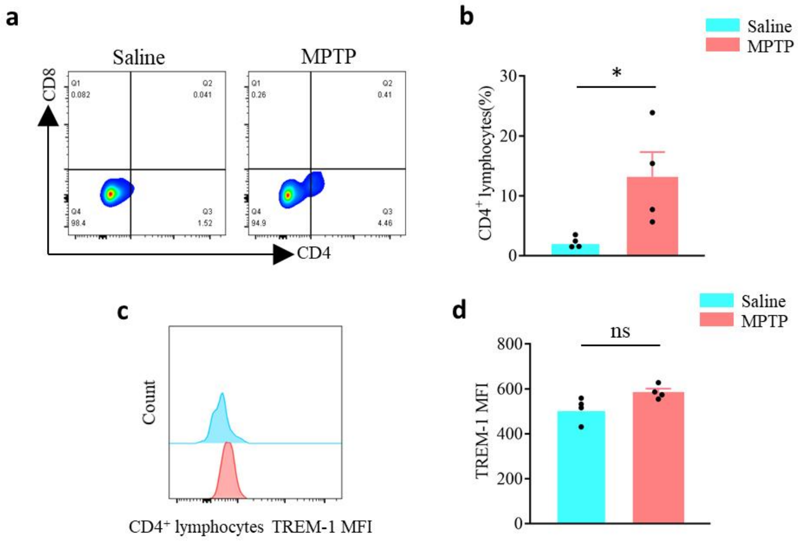

Fig. S15 CD4^{+} T-lymphocyte infiltration in SNpc. (a) Plots identify CD4^{+} T-lymphocyte. (b) Percentages of CD4^{+} T-lymphocyte detected in SNpc by flow cytometry (n = 4) (c) Representative histograms of TREM1 expression on CD4^{+} T-lymphocyte. (e) CD4^{+} T-lymphocyte TREM-1 MFI in the SNpc (n = 4). The data are presented as the mean ± SEM. (*$P < 0.05$, **$P < 0.01$, or ***$P < 0.001$ by Student's t test).
