# Supplementary material for: Infiltrating peripheral monocyte TREM-1 mediates dopaminergic neuron injury in substantia nigra of Parkinson’s disease model mice
Source: Cell Death Dis. 2025 Jan 14;16(1):18. doi: 10.1038/s41419-025-07333-5 (PMC11733277; doi:10.1038/s41419-025-07333-5)

Figure1

F

TH

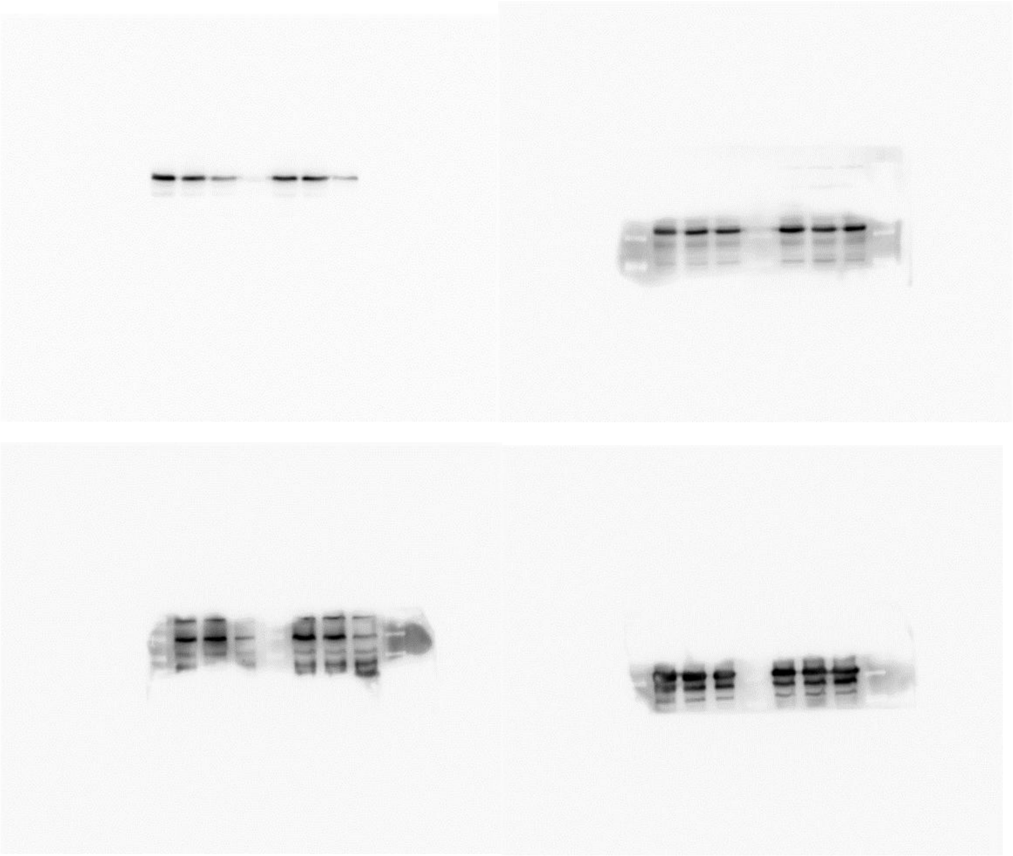

Figure3

d

TREM-1

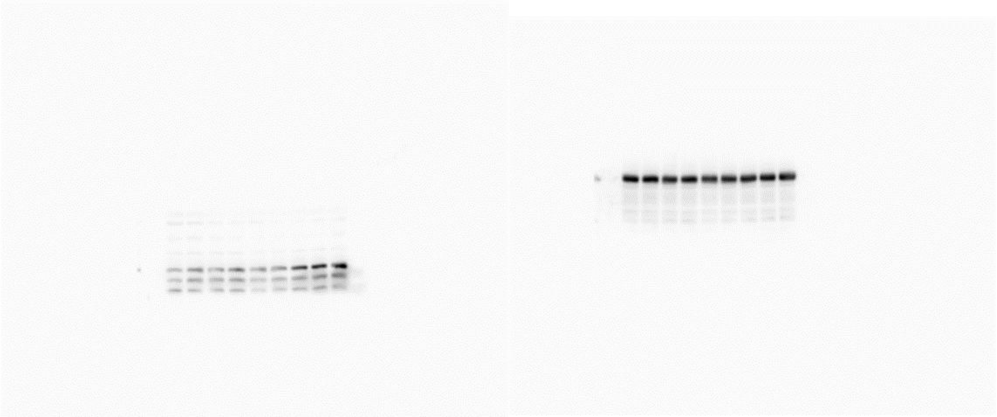

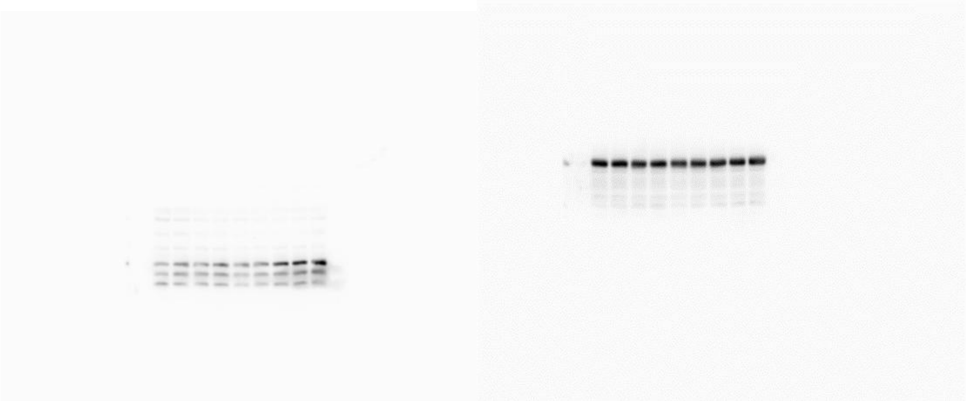

Figure4

a IL -6

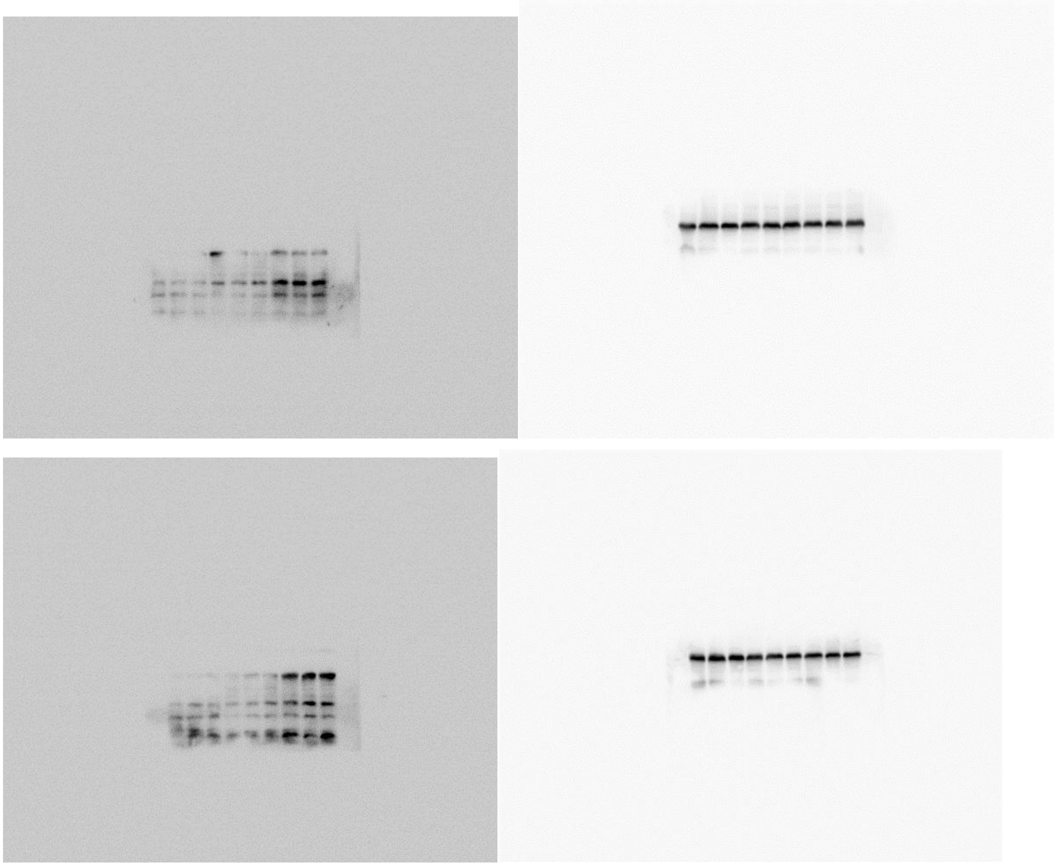

b IL1-β

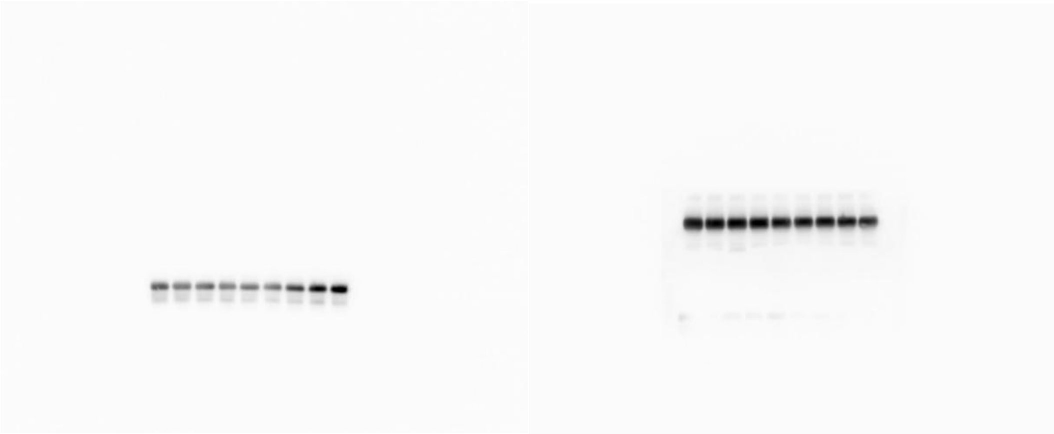

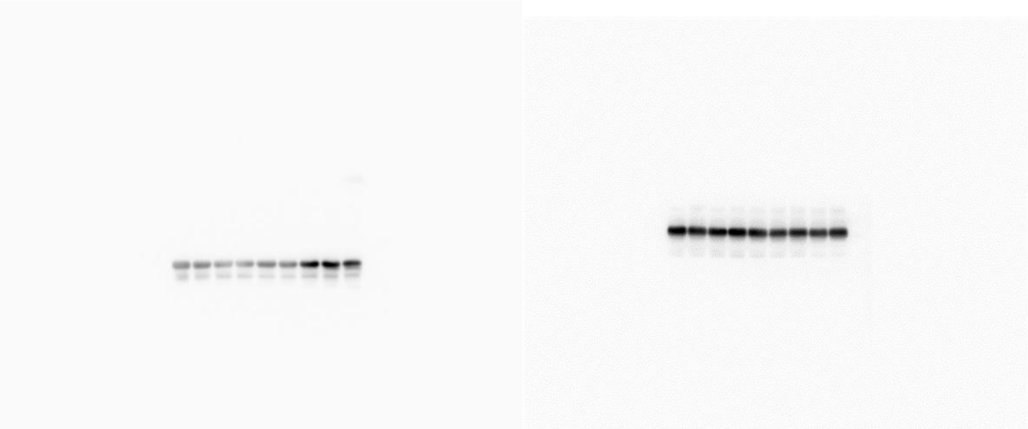

TNF-α

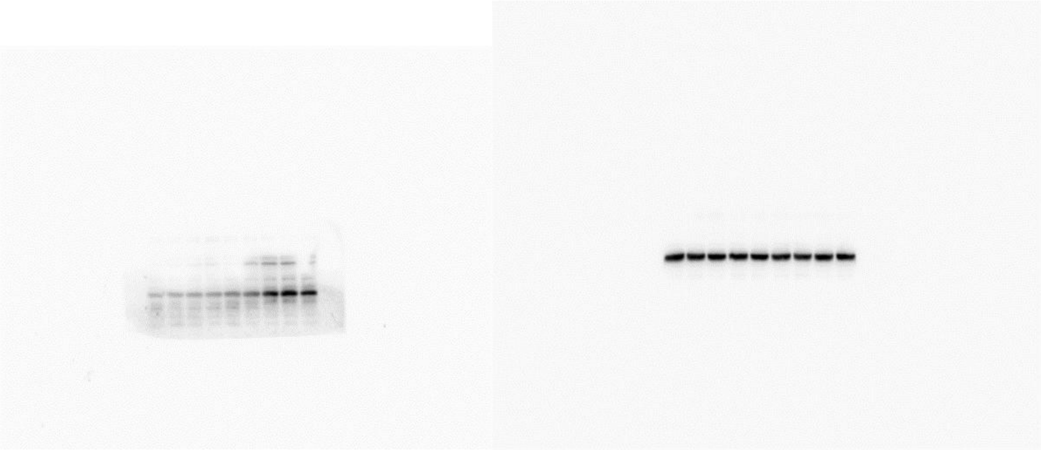

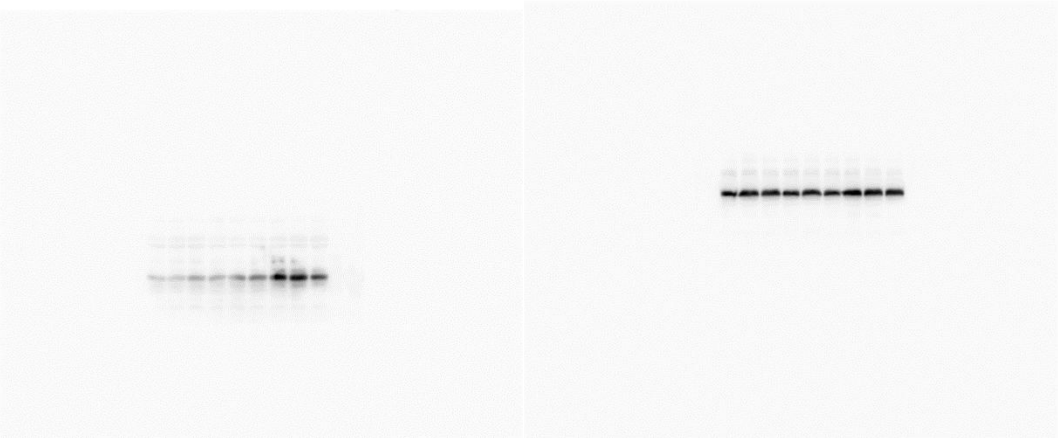

d

TREM-1

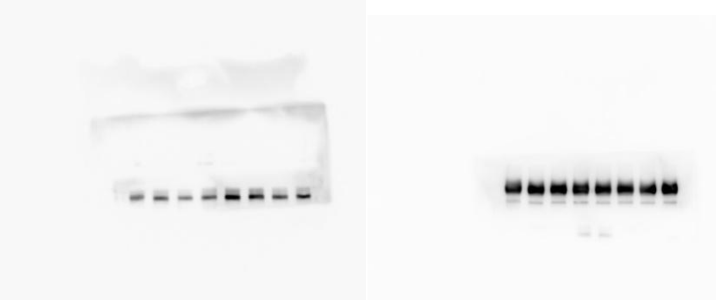

g

IL-6

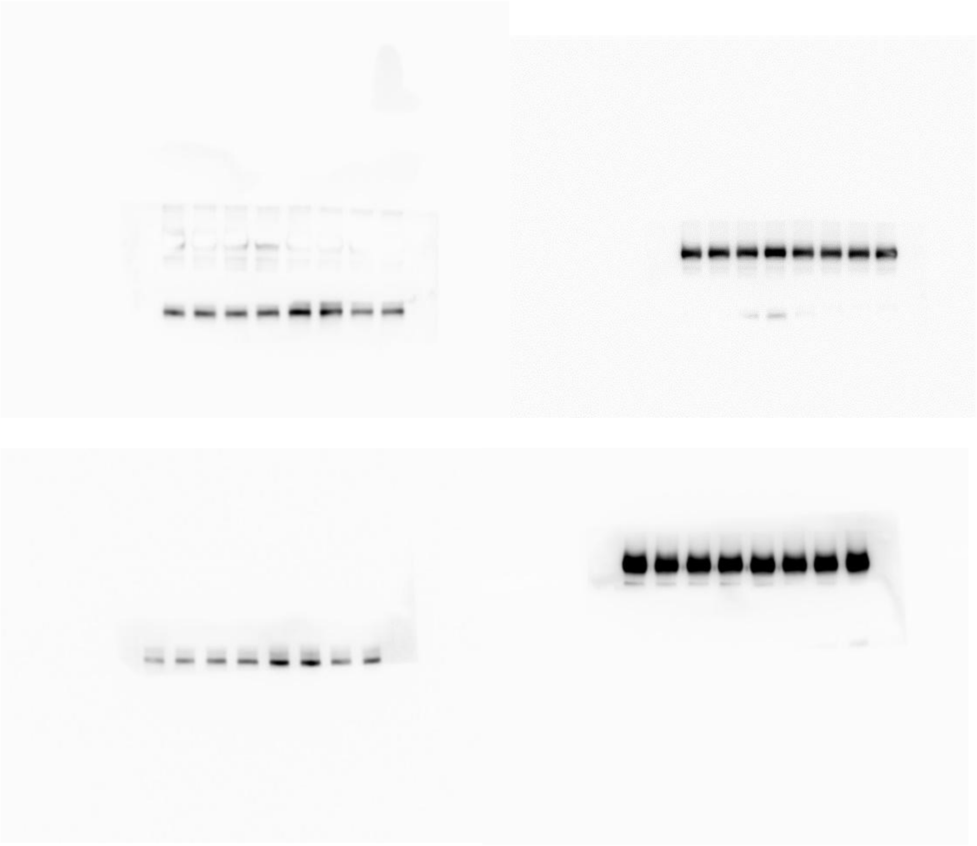

Il-1β

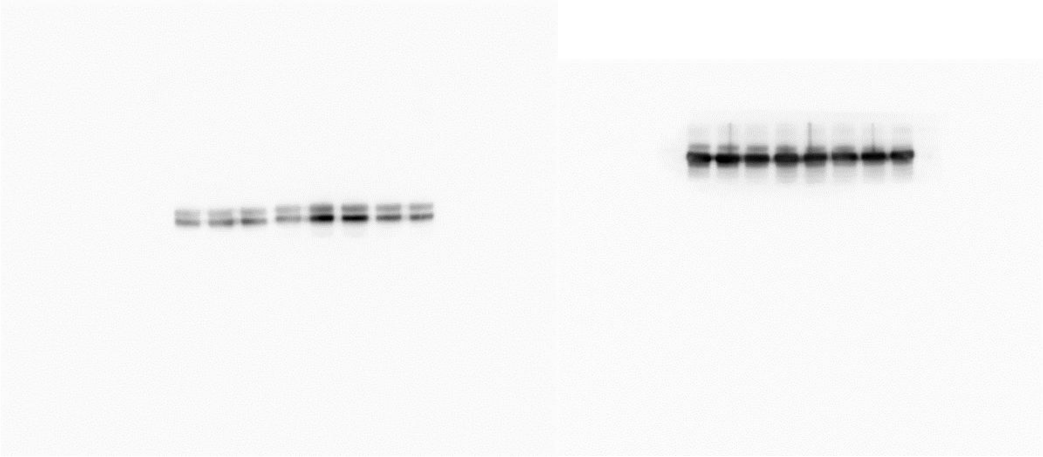

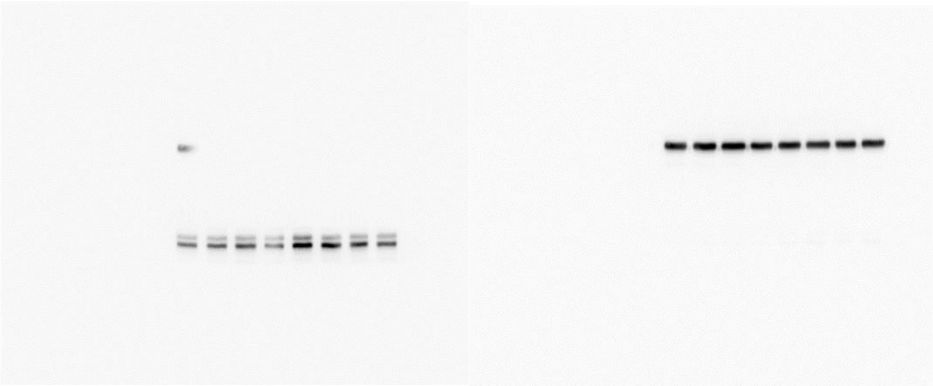

TNF-α

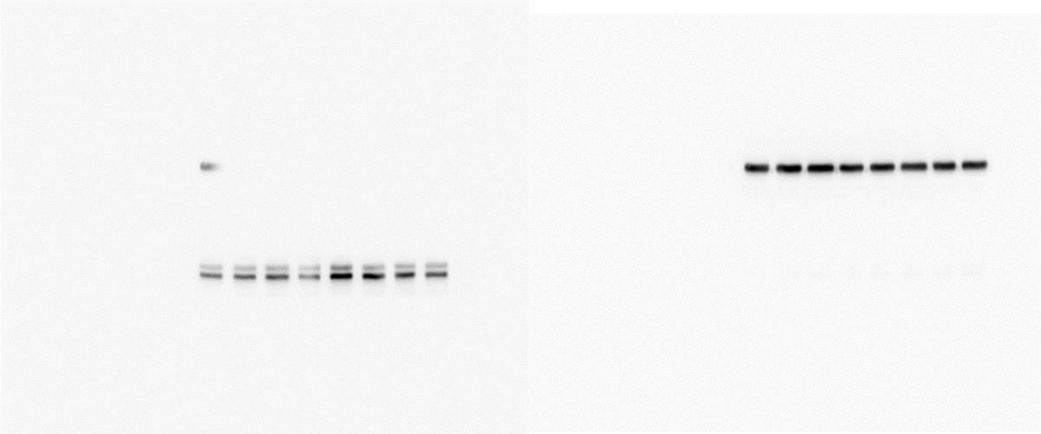

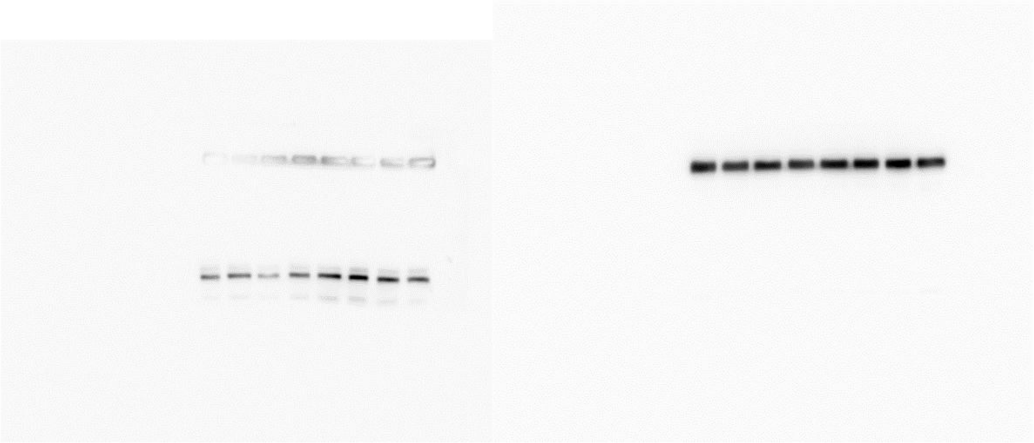

Figure 5

a

TREM-1

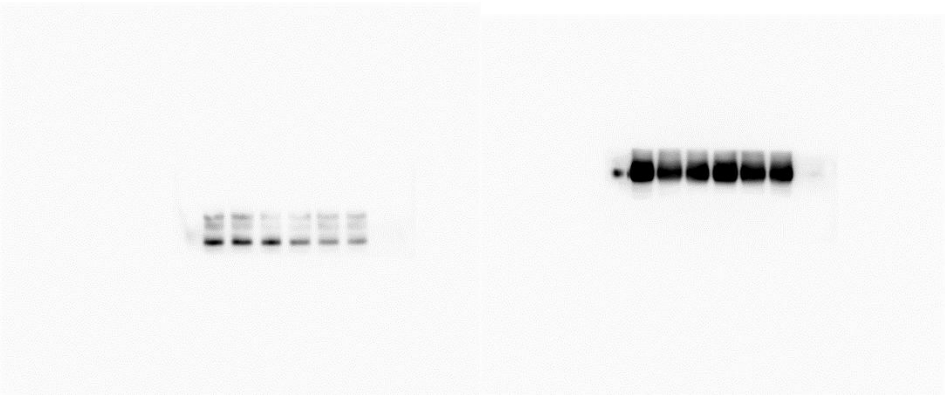

e

TH

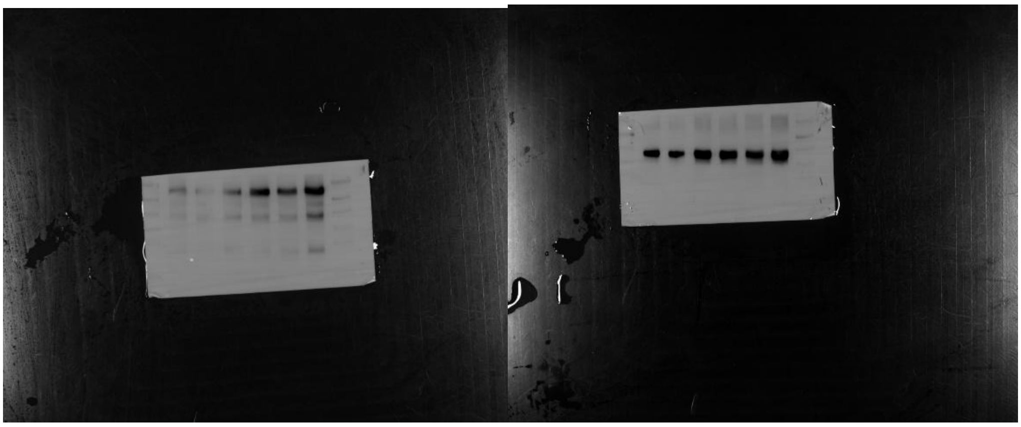

n

IL-6

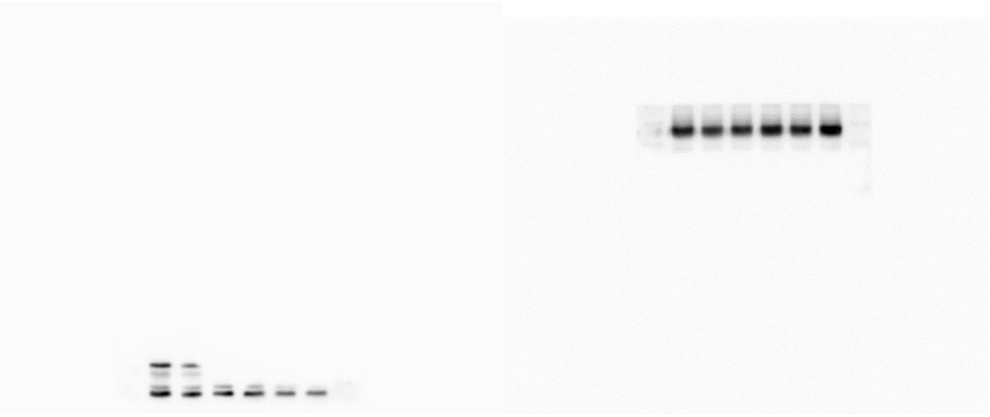

TNF

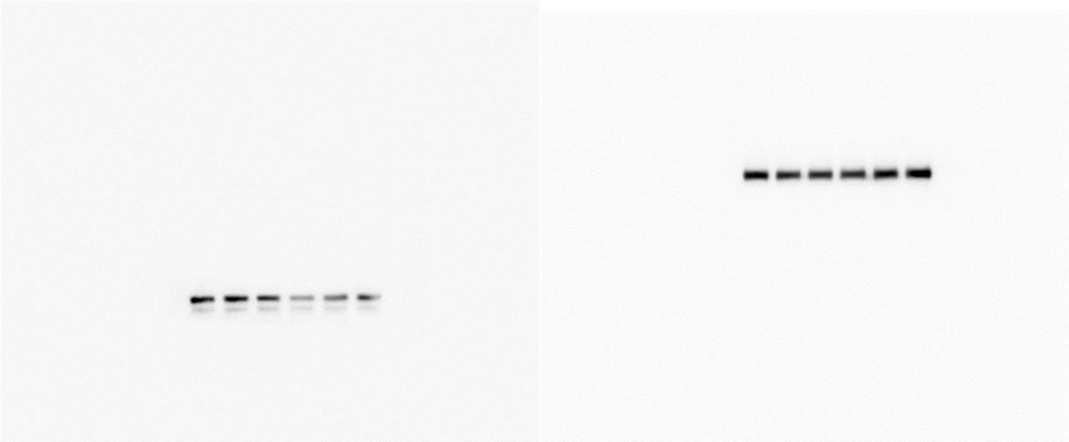

Figure 6

f

TREM-1

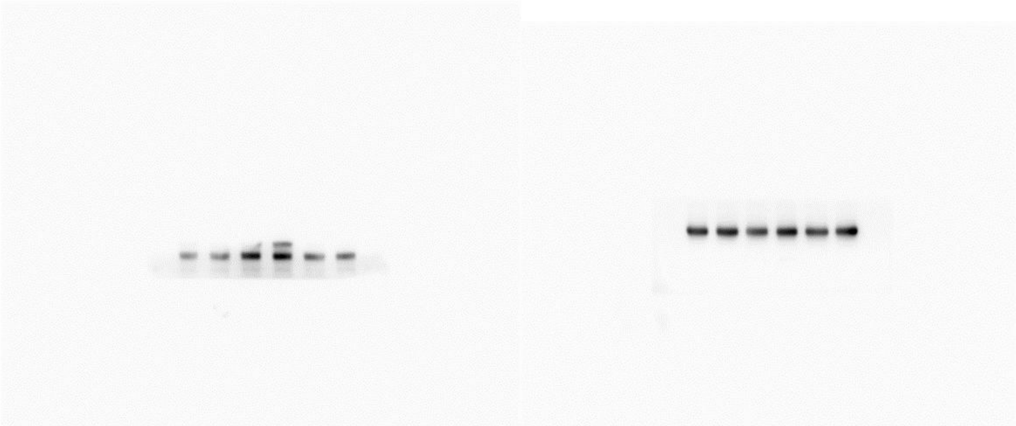

I

IL-6

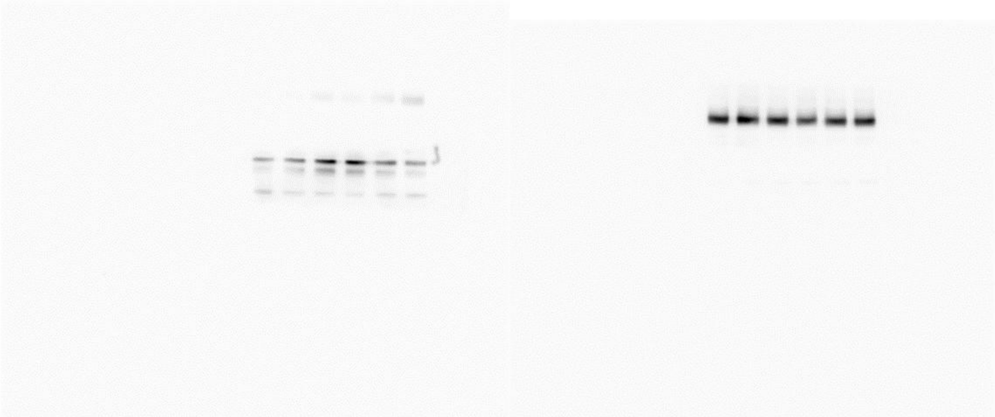

IL-1β

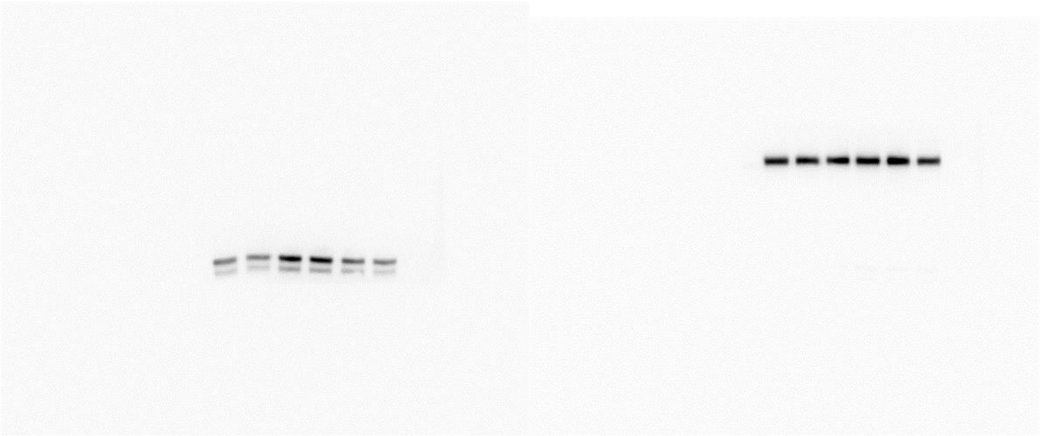

TNF-α

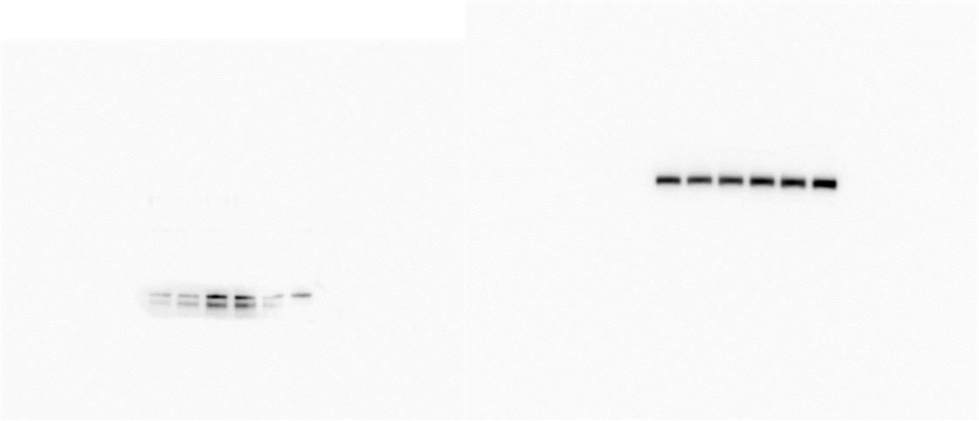

Figure 7
e
TREM-1

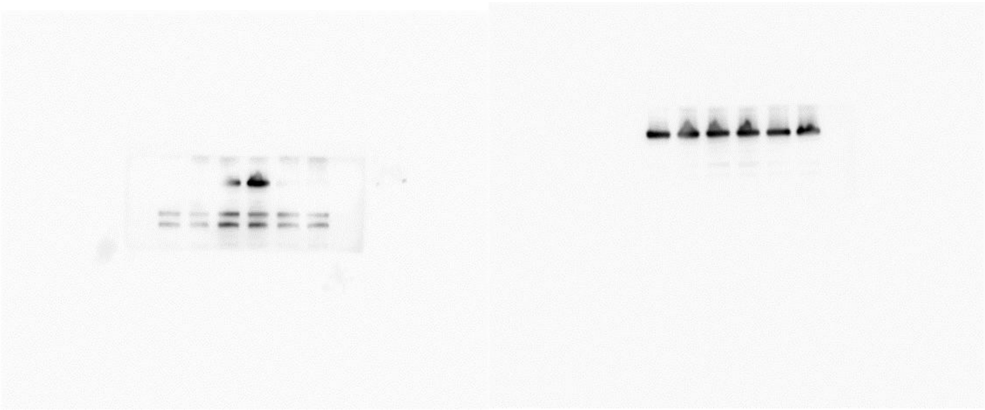

h
IL-6

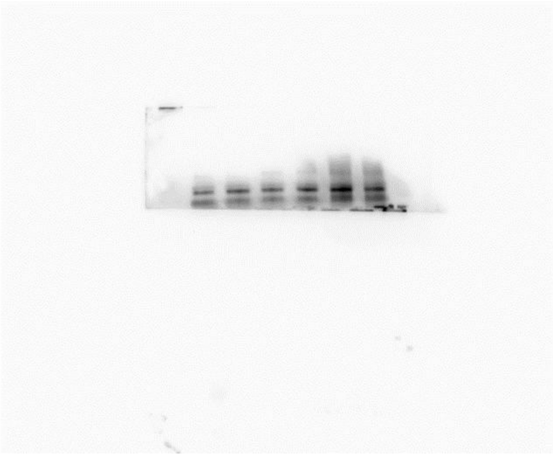

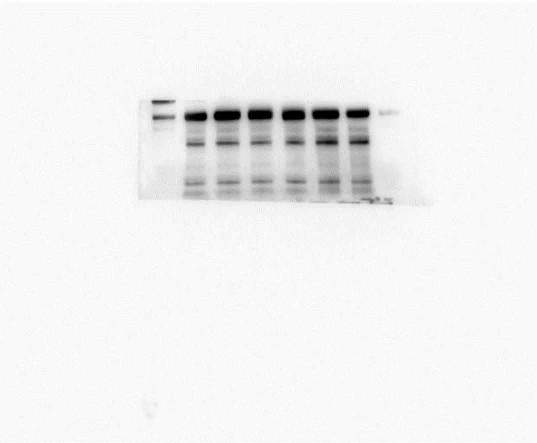

IL-1

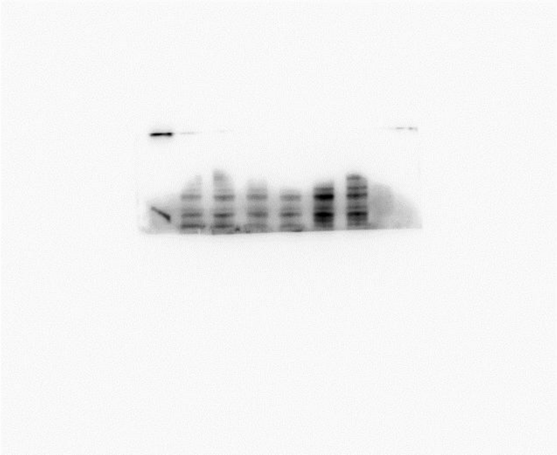

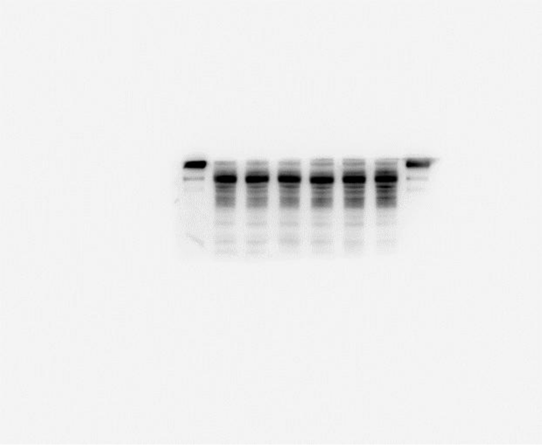

TNF-α

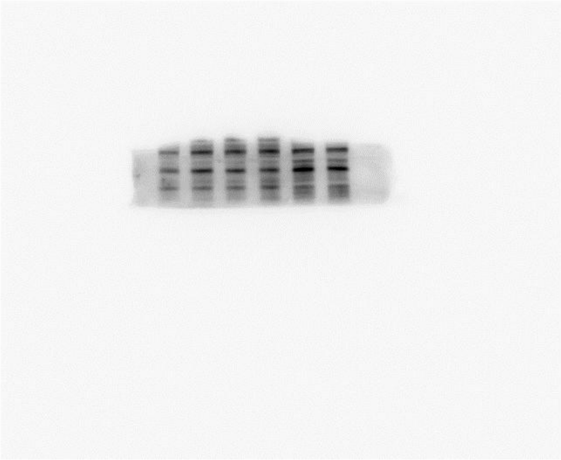

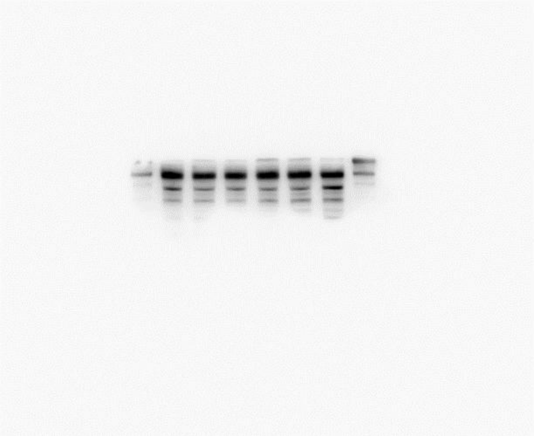

Supplement: Supplementary file 2 — Full gel and blot images [file 41419_2025_7333_MOESM2_ESM.pdf]
